# A Review of Chloride Penetration of Recycled Concrete with Enhancement Treatment and Service Life Prediction

**DOI:** 10.3390/ma17061349

**Published:** 2024-03-15

**Authors:** Yuanzhan Wang, Jing Liao, Baohua Zhang

**Affiliations:** 1 State Key Laboratory of Hydraulic Engineering Simulation and Safety, Tianjin University, 135 Yaguan Road, Jinnan District, Tianjin 300072, China; yzwang@tju.edu.cn; 2 Tianjin Research Institute for Water Transport Engineering, M.O.T., 2618 Xingang 2nd Road, Binhai New District, Tianjin 300000, China

**Keywords:** recycled coarse aggregate concrete, chloride penetration, enhancement method, modification efficiency, service life prediction

## Abstract

The application of recycled coarse aggregate (RA) in structural concrete can save non-renewable resources and reduce land occupation. Developing comprehensive knowledge of chloride penetration and service life modeling of recycled coarse aggregate concrete (RAC) is a prerequisite for practice. However, compared with the natural aggregate concrete (NAC), the inferior durability performance, especially chloride penetration resistance, of RAC hinders its application in structural concrete. Therefore, many RAC performance enhancement methods have been proposed. This paper presents a holistic review focused on the chloride penetration of RAC with/without enhancement methods and service life prediction. The current RAC performance enhancement methods are introduced. The improvement effect of the corresponding enhancement methods on the chloride penetration resistance of RAC are discussed and analyzed in turn. Based on the reviewed data on the chloride diffusion coefficient, the modification efficiencies of assorted enhancement methods are summarized. With the hope of promoting RAC application in structural concrete, the current literature on chloride-ingress-based service life prediction for RAC is also overviewed. In addition, the typical influencing factors on chloride transport properties are also discussed, i.e., RA quality. It can be concluded that enhancement techniques can effectively improve the chloride penetration resistance of RAC. The old mortar enhancement or removal methods can improve the chloride penetration resistance by 15–30%, depending on the specific treatment measures. The modification efficiency of the modifier material depends on the specific type and content of the incorporated substance, which ranges from approximately 5% to 95%. The estimated service life of RAC structures decreases with the increasing RA replacement ratio. Finally, concluding remarks are provided concerning future research on the chloride transport behavior of RAC.

## 1. Introduction

With the advancement of urbanization, concrete has become widely utilized in the construction industry, resulting in dramatically increased manufacturing of concrete products. More specifically, it is estimated that approximately 30 billion tons of concrete are produced annually worldwide [1]. Concrete manufacturing activities consume 50% of total natural raw material, 40% of total energy, and generate 50% of total waste [2]. For example, the huge demand for aggregates has led to over-extraction of natural aggregates. Additionally, building demolition activities and natural disasters result in a large amount of construction and demolition waste (CDW), which may cause serious environmental problems, i.e., air, water, and soil pollution, as well as land occupation. It is reported that concrete and brick waste account for about 70% of CDW [3]. Concerns about the sustainability of the construction industry have prompted the reutilization of waste concrete. According to the literature [4], aggregates account for 50–90% of waste concrete, and recycling waste concrete into aggregates will greatly contribute to CDW reduction. In this context, the recycling of recycled coarse aggregate (RA) will not only control natural resource consumption and environmental pollution, but also promote a circular economy, in line with the concept of sustainable development in the construction industry.

As early as the 1970s, Japan started to build factories to collect RAs [5]. After World War II, Europe and some developed countries began to study the performance of RAC [6]. Until the 1990s, with the development of urbanization, China also explored the feasibility of using RA to manufacture concrete [7]. Nonetheless, it was not until the 2000s that RA really began to see practical application as a sustainable material [8]. During the past decades, research into recycled aggregate concrete has emerged. The relevant research mainly includes the microscopic, mechanical properties and durability of RAC. Ridzuan et al. [8] preliminarily analyzed the feasibility of applying RAC in medium-strength concrete structures by demonstrating that RAC achieved comparable compressive strength to NAC. Poon et al. [7] found that RAC prepared with RA derived from high-performance concrete achieved strength similar to that of NAC. They also found that the parent concrete not only affected the old mortar, but also affected hydrate product development in new ITZs in RAC. Similarly, Ajdukiewicz et al. [9] found the RAC prepared with RA derived from strong concrete achieved similar compressive strength and stress–strain curves to those of NAC, while the tensile strength and failure bond stress were 10% and 20% lower, respectively. They claimed that manufacturing high-performance concrete with RA can achieve both environmental and economic benefits. Also, in a study by Akita et al. [10], it was found that RAC with a 5% lower w/c ratio achieved similar compressive strength to that of NAC, while the tensile strength was considerably decreased. Poon et al. [6] investigated the effects of different RA contents and moisture conditions on RAC’s compressive and splitting tensile strength and found that RAC with a 50% air-dried state RA replacement ratio showed similar strength to that of NAC. By a series of comparison tests, Tabsh and Abedlfatah [11] found that the addition of RA caused a 10–25% loss in RAC’s strength compared with NAC.

Based on the above research results, it can be concluded that replacing NA with RA is a promising method for manufacturing concrete, even high-performance concrete, from the perspective of mechanical properties. Apart from mechanical properties, durability is another significant aspect that must be explored before applying RAC in practical engineering. The durability and the time-dependent chloride transport properties of RAC have been extensively studied in recent years. Olorunsogo et al. [12] concluded that RAC was more susceptible to oxygen permeability, ion diffusion, and water absorption by detecting durability indexes. Thomas et al. [13] reported that the difference between the durability indexes of NAC and RAC with the same w/c ratio increased with increasing w/c ratios. They also provided recommended mixtures for RAC in aggressive environments. Levy et al. [14] reported that a replacement ratio of 20% for RA to NA made RAC show similar or sometimes better durability than NAC in terms of carbonation, porosity, and water absorption. By conducting durability tests and environmental impact assessment, Henry et al. [15] successfully prepared RAC with low-grade RA with similar or better durability and lower CO_2_ emissions compared with NAC. Abbas et al. [16] investigated the freeze–thaw resistance, chloride penetration, and carbonation properties of RAC prepared with the equivalent mortar volume method (EMV). The results indicated that, compared with RAC designed using normal methods, the RAC designed by EMV was more durable and met the current requirements for concrete used in aggressive environments. Richardson et al. [17] explored the freeze–thaw properties of RAC by detecting the weight and compressive strength loss. The results indicated RAC with treated, good-quality RA showed 68% higher freeze–thaw resistance than NAC. Bravo et al. [18] determined the detrimental influence of RA on the water absorption, carbonation depth, and chloride diffusion coefficient of RAC. They found RA compositions varied with the collecting areas, which in turn affected the durability of RAC. Debieb et al. [19] investigated the mechanical performance and durability of RAC containing contaminated RA. They found that although the strength, porosity, and freeze–thaw resistance were free of influence by contaminated RA, the corrosion risk of reinforcement in such RAC should be taken into consideration. Since chloride-induced corrosion dominates the long-term durability of reinforced concrete (RC) structures, some scholars have reported relevant research results on chloride penetration and service life prediction for RAC. For example, Villagrán-Zaccardi et al. [20] investigated chloride penetration and binding capacity in RAC. Lian et al. [21] conducted experimental and theoretical analyses of chloride ion transport in RAC under wet and dry cycling. Srubar [22] presented and implemented a stochastic service-life model for chloride-induced corrosion of RC structures with RAC and analyzed the effects of RA types, RA replacement ratios, and initial aggregate chloride concentrations.

Based on the large number of existing studies, in order to further understand RAC characteristics and assist designers and scholars in quickly and clearly grasping relevant research results, development levels, and technological trends, some review papers have been published. For example, Letelier et al. [23] presented a review of the effect of coarse types on the mechanical properties of RAC. Kim [24] reviewed the literature on the mechanical properties of RAC from the perspective of the influence of RA quality. They elaborated the relationship between RA quality and the compressive, tensile, and flexural strength, elastic modulus, and drying shrinkage of RAC. Wang et al. [1] reviewed the literature on the compressive strength and elastic modulus of RAC prepared with waste concrete and brick coarse aggregate in the past 40 years. Zheng et al. [25] reviewed the literature on the mechanical performance of RAC after exposure to high temperatures. Deresa et al. [26] presented a review of the flexural and shear response of RC beams with RAC from a structural perspective. Bahraq et al. [27] presented a comprehensive review of the mechanical properties and durability aspects of RAC. They comprehensively elaborated RAC’s strength, stress–strain curves, elastic modulus, Poisson’s ratio, bond strength, and fracture energy, as well as its dry shrinkage, freeze–thaw, carbonation, sulfate, chloride penetration, air permeability, and fire resistance. Shahjalal et al. [28] presented a holistic review of the durability of RAC, examining impermeability, chloride penetration resistance, carbonation resistance, frost resistance, and alkali–silica reactions. Malazdrewicz et al. [29] provided an overview of existing studies on the durability of self-compacting RAC, examining chloride permeability, carbonation behavior, and freeze–thaw resistance.

In addition, due to the drawbacks of RAC in terms of mechanical properties and durability compared with NAC [30,31,32,33,34,35], RA is mainly used as a base filler for pavement engineering. In recent years, many scholars have focused on RAC performance-improving technology intended to further improve the utilization rate of RA [36]. Al-Bayati et al. [37] investigated the influence of HCl and acetic acid presoaking on the rubbing removal rates of old mortars. The results indicated the old mortar was prone to removal after acid presoaking. RA surface treatment with nanoparticles [38,39,40,41], polymer emulsions [41,42,43,44], and pozzolanic materials [34,40,45,46,47,48] can effectively improve RA performance by refining its porosity and densifying its microstructures. During the last decade, an accelerated carbonation technique has been proposed and intensively studied by many researchers to improve the mechanical performance and durability of RA [38,42]. As noted by refs. [49,50,51,52,53], the incorporation of supplementary cementitious materials (SCMs) is also a promising technology for obtaining more durable RAC structures. In addition, different mixing methods also have a certain degree of influence on the porosity of RA [16]. There is a body of literature reporting the properties of RAC after enhancement treatment [54].

In summary, (i) although the chloride penetration properties of RAC have been summarized in some review papers, they mainly focus on durability index measurement values at the material level and do not consider long-term durability deterioration modeling. (ii) Although there is a body of literature concerning service life estimation for RAC structures, a holistic review is still lacking. Undoubtedly, one should obtain a thorough knowledge of service life prediction for RAC, which addresses how long a RAC structure will fulfill its intended function. (iii) The above RAC-improving techniques make it possible for RAC to be utilized in structural concrete with satisfactory mechanical performance and durability. Enhancement techniques inevitably change RAC’s microscopic pore structure, chloride-binding ability, etc., which has a certain effect on the chloride transport behavior of RAC structures [55]. It is necessary to understand these key properties to control the deterioration behavior of RAC structures.

A large volume of research results has been obtained concerning the mechanical properties, chloride ion transport characteristics, and durability of RAC. In recent years, improving the chloride penetration resistance and the corresponding durability design of RAC has become an important topic, and abundant research results have been obtained. This paper provides a holistic review of the relevant research on the chloride transport properties of enhanced RAC structures and corresponding durability design aspects, which can provide a reference for further research and engineering applications of the research results. First, a brief review of the current research on performance enhancement techniques is presented. Then, the modification efficiencies of various enhancement methods with specific treatment conditions on the chloride penetration of RAC are analyzed and discussed in detail. In addition, the influences of the RA replacement ratio, RA quality, and coupled damage of freeze–thaw cycling or loading on chloride penetration are introduced, and studies on the chloride ion-binding capacity of RAC are reviewed. With the purpose of promoting RAC application in structural concrete, the authors also overview the current research on service life prediction at the elemental level. Furthermore, the conclusion of this review and feasible future research points are presented in the last section.

## 2. Enhancement Methods for RAC

**Figure 1:**
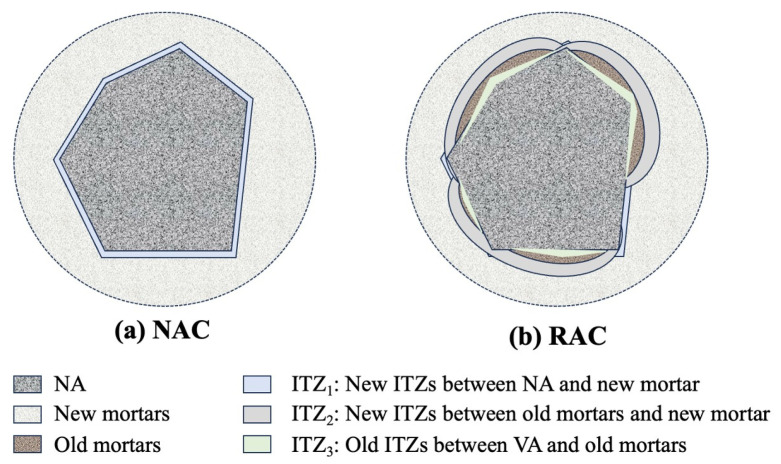
(**a**) Composition and structure schematic diagram of NAC; (**b**) Composition and structure schematic diagram of RAC.

In order to understand the effect of enhancement methods on chloride permeability in RAC, it is necessary to understand the difference between ion transport channels in RAC and NAC. As is well known, the meso-three phases of NAC consist of mortar, coarse aggregate, and an interfacial transition zone (ITZ). However, the mesoscopic composition in RAC is different from NAC. RA is composed of NA and adhered mortar, which is part of the mortar in parent concrete, as shown in Figure 1. Due to the existence of RA, the ITZs in RAC can be categorized into three types: (i) ITZ_1_: lying between the virgin aggregate and new mortar; (ii) ITZ_2_: lying between the old mortar and new mortar; (iii) ITZ_3_: lying between the old mortar and NA. Thus, mesoscopic RAC is composed of NA, new and old mortar, and three categorizes of ITZs, as shown in Figure 1. The high chloride ion permeability of RAC compared with NAC can be attributed to the fact that: (i) RA consists of 65–70% NA and 30–35% old mortar [56,57] (NA is a dense material that is nearly impermeable, while mortar is permeable due to the existence of pores, cracks, etc.). (ii) Due to the higher water absorption of RA, the w/c ratio in ITZ_2_ in RAC is higher than ITZs in NAC, which results in the ITZ_2_ in RAC having a larger width and lower micro-hardness [58]. (iii) The higher mud content in RA may result in ITZ_1_ in RAC having a larger width and lower micro-hardness [58]. (iv) The total length of all ITZs in RAC is 60–70% larger than that in NAC [58]. It is worth noting that the high content of acicular calcium hydroxide crystals is the main reason for the low micro-hardness and high chloride ion penetration [59].

Based on the reasons for the inferior performance of RAC, the following RAC enhancement methods have been developed: (i) surface mortar modification, including two mechanisms for sealing with coating and filling with micro-filler; (ii) old mortar removal, including mechanical friction removal, high temperature removal, chemical immersion, etc.; (iii) mineral admixture, nanomaterial, or fiber addition to improve the whole performance of RAC to compensate for the defects brought by RA.

In the following section, the above-mentioned RAC’s performance enhancement methods are briefly introduced in turn.

### 2.1. Pre-Treatment of RA

#### 2.1.1. Surface Mortar Modification

Slurry wrapping treatment and polymer impregnation treatment, which treat the RA surface with cement slurry, pozzolana slurry, phosphate solution, calcium metasilicate and polymer solution, etc., are effective methods for mortar modification [5]. Soaking and spraying are two common treatment methods. These methods do not require large equipment and are easily applied in practical engineering on a large scale at low cost. The improvement mechanism varies according to the specific solution used. Some solutions physically fill the pores and cracks of mortar and aggregate, and some solutions chemically react with mortar or aggregate. The specific mechanisms, implementation measures, and improvement effects reported in the literature are summarized in Table 1. It is worth noting that although the water absorption of RA is reduced by some treatments, other properties of RAC such as strength are negligibly unchanged [35,38,42,60,61,62].

Carbonization treatment, as another promising method for mortar enhancement, has been extensively investigated during the past decades [67]. Calcium hydroxide (CH) with high CO_2_-reactivity is prone to precipitating calcium silicate in mortar [68], which increases the total volume of the mortar by 3%, thus densifying the mortar and improving the properties of RA [69]. Actually, CH is prone to natural reaction during the service of the parent concrete. It is therefore necessary to provide an additional source of calcium to generate enough precipitates to fill the pores [70]. This provides a two-pronged approach for high-calcium wastewater treatment. Also, solidification of carbon dioxide is beneficial for mitigating the environmental problems caused by greenhouse gas emissions. Although studies have shown that carbonization reduces the total porosity of RA by approximately 30% [70,71], water absorption by 5–45% [67], and electrical conductivity and chloride ion permeability by approximately 70% [72], excessive carbonation may cause adverse effects, i.e., lower pH values and chloride thresholds for corrosion initiation [73]. For this reason, alkaline SCMs such as red mud (RM) may counteract this negative effect when applied in carbonated RAC [49], which requires further investigation. At present, the principal mainstream carbonization methods are the tentative method and quick method pressurized standard carbonation [56,57], gas–solid carbonation (PC) [74,75], flow-through gas–solid carbonation (FC) [76,77,78], wet carbonation (WC) [79], etc. Although a large number of studies have been reported on the influence of carbonization conditions (such as temperature, humidity, etc.) on the carbonization rate [69,80,81], the relationship between the phase of the carbonization product, carbonization conditions, and carbonization rate has not been fully studied.

#### 2.1.2. Old Mortar Removal

It has been reported that the adverse effect of old mortar on RAC performance is proportional to RA content [82]. This inspired a series of studies on methods for detaching old mortar from RA, which embrace mechanical friction [83,84,85], conventional heating [82,83,86,87], microwave-assisted beneficiation [83,88,89], and acid soaking beneficiation [37,48,49,62].

Mechanical friction technology is based on the mechanism of increasing the frictional action between aggregates while minimizing the degree of aggregate breakage [83]. Conventional heating techniques are based on the mechanism of thermal stress difference between virgin aggregate particles and adhered mortar induced by thermal expansion [86]. Immersing the heated RA in cold water immediately after heating is proposed as a practical method for improving the removal rate [82]. However, the heating method is not advised due to its high energy consumption and CO_2_ emissions [62]. The principle of microwave-assisted beneficiation is based on the internal mechanical stresses generated between virgin aggregate and mortar under the same microwave conditions [83,88]. The embrittlement of mortar, especially the embrittlement of ITZ, forms a weak zone prone to brittle fracturing [88]. Tam et al. [90] studied the effects of HCl, H_2_SO_4_, and H_3_PO_4_ in removing attached mortar in RA. They concluded that the water absorption and mechanical properties of RA were improved by acid treatment. The RAC with low-HCl concentration solution-treated RA showed higher compressive, splitting tensile, and flexural strength by 6%, 8%, and 3% compared to normal RAC [91]. However, the existence of chlorides and sulfates in RA may cause durability issues, and the disposal of washing water may pose environmental issues. Thus, Al-Bayati et al. [61] provided another alternative by demonstrating the excellent removal effect of acetic acid. Recently, a new environmentally friendly acid treatment processing was proposed, which holds promise for recovering acetic acid, sequestering carbon, and producing waste cement for soil stabilization [37].

### 2.2. Mineral Admixture, Nanomaterial, or Fiber Addition

**Figure 2:**
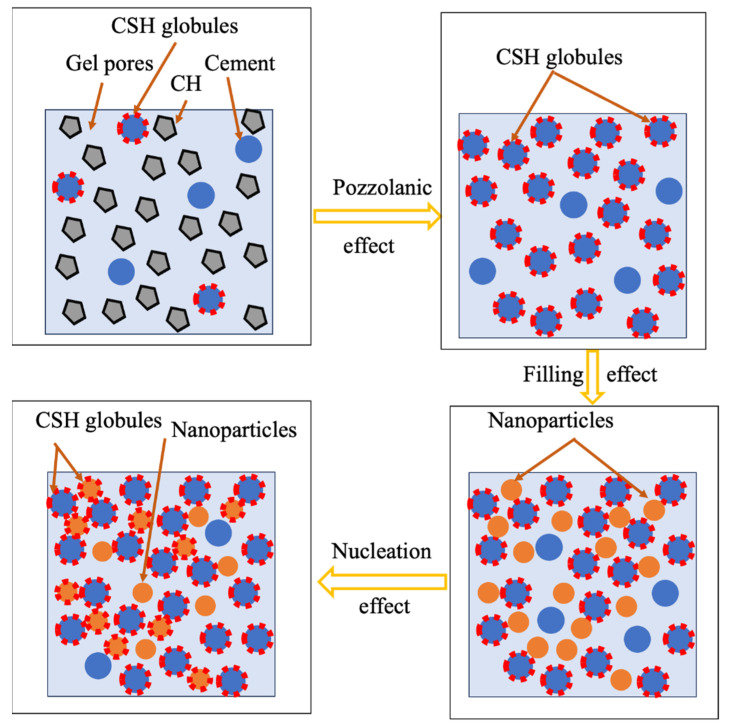
Schematics of the pozzolanic effect, filling effect, and nucleation effect.

The incorporation of SCMs as an efficient way to improve performance, extend service life, and reduce the environmental impact of NAC structures [49,50] is also applicable to RAC [5]. Coupled with increasing interest in SCMs as partial replacements for binders in RAC, research on RAC with SCMs has overwhelmingly concerned conventional SCMs such as FA and granulated blast furnace slag (GGBFS) incorporated into novel cements developed from byproducts and waste glass, such as bagasse ash (BA), glass powder (GP), etc. [51]. Current varieties of SCMs are mainly divided into soluble siliceous, aluminosiliceous, and calcium aluminosiliceous powders [51]. Furthermore, since nanomaterials can improve the reaction kinetics and the physical properties of cement due to their higher specific surface area, they have been used in RAC for performance improvement [92]. Additionally, fiber materials with high toughness and tensile strength also serve to improve RAC properties [5]. The main mechanisms of material addition for RAC improvement can be categorized into filling effects, pozzolanic effects, nucleation effects, and bridging effects [92], as shown in Figure 2. The pozzolanic effect indicates that the active components of a pozzolanic substance react with CH to produce reaction products such as CSH, calcium aluminate hydrate, or calcium sulfoaluminate hydrate [92]. The filling effect indicates that micro-fillers fill the pores in concrete [92]. The nucleation effect means that some substances, such as nanomaterials, can provide additional nucleation sites to create conditions for the crystal growth of hydration products [93]. The fibers form a three-dimensional network structure in the concrete, which bridges cracks when the concrete is subjected to external forces, preventing it from continuing to expand [36].

## 3. Chloride Penetration

### 3.1. Mechanism, Test Methods, and Corresponding Theories

It is generally believed that chloride ions enter concrete mainly through diffusion, capillary adsorption, electromigration, etc. [94]. Diffusion is the dominant transport method in both saturated and non-saturated concrete [95]. When there is a concentration gradient between the external solution and the pore liquid, chloride ions will naturally diffuse from the outside into the continuous liquid phase inside the concrete [96]. For concrete undergoing wetting and drying cycles, the capillary adsorption is the dominant transport method at the surface region of porous materials [94]. When water comes into contact with a dry surface, the capillary action causes the water carrying chloride ions to be quickly sucked into the concrete. The region affected by liquid evaporation and capillary suction is called the convection area [96]. The electromigration of ions is driven by the electric potential gradient. In the absence of an external voltage, electromigration can be ignored [97].

Since chloride erosion is one of the most serious causes of reinforcement corrosion, testing methods for chloride transport have become the focus of many studies and specifications. In general, these test methods fall into four main categories: (i) microscopic characterization (i.e., porosity, micromorphology analysis); (ii) indirect physical tests (i.e., water absorption, resistivity); (iii) electromigration tests (i.e., the rapid chloride migration test (RCMT), the rapid chloride permeability test (RCPT)); (iv) direct tests (i.e., the artificial natural diffusion simulation test, field tests). Methods (i) and (ii) are taken as the qualitative analysis means for chloride resistance for concrete, while Methods (iii) and (iv) are widely used to describe/predict chloride transport behavior mathematically. Based on the latter two test methods, two types of chloride ingress models were developed: (i) the analytical solution of Fick’s second law based on the assumptions in Equations (1) and (2) [98]; (ii) the governing equation established based on Fick’s first law and the mass conservation equation in Equations (3) and (4) [94]. The two methods have their own advantages. The former is widely used for its engineering practicability, and is included in durability specifications in many countries. Using natural diffusion testing, chloride profiles can be obtained by grinding concrete power and chloride concentration titration at different ages [97]. The free chloride ion concentration and total chloride ion concentration can be obtained by titration with distilled water and nitric acid solution, respectively [97]. Then, the apparent chloride diffusion coefficient, *D*, and surface chloride concentration, *C*_s_, can be obtained by fitting the chloride profile to Equation (2). The regression analysis of *D* and *C*_s_ over a series of ages based on Equations (5) and (6) can establish the empirical relationships of *D* and *C*_s_ with exposure time. In this way, the long-term performance of chloride ion transport can be predicted based on short-term data. The chloride diffusion coefficient at a fixed age can also be determined by acceleration test methods, e.g., chloride migration testing, rapid chloride migration testing (RCMT), etc. For example, according to [98], the *D*_0_ in Equations (2) and (5) can be replaced by *D*_RCM_. The latter is commonly used with moisture sorption isotherms or binding isotherms, etc., to take convection or binding into consideration. The modified equations can be solved by a numerical method [96]. However, the latter is more complicated to solve and apply.

(1) $$\frac{𝝏C}{𝝏x}=D\frac{{𝝏}^{2}C}{𝝏x^{2}}$$

(2) $$C(x,t)=C_{0}+(C_{s}-C_{0})\left[1-erf\left(\frac{x}{2\sqrt{Dt}}\right)\right]$$

(3) $$J=-D_{cl}grad(C_{f})$$

(4) $$\frac{dC_{t}}{dt}=-div(J)$$

(5) $$D(t)=D_{0}{\left(\frac{t_{0}}{t}\right)}^{m}$$

(6) $$C_{s}=C_{s\max}(1-e^{-rt})$$

Here, *C* denotes the chloride content (% by mass of concrete); *D* denotes the apparent chloride diffusion coefficient; *t* denotes age; *C*_0_ denotes the initial chloride content; *C*_s_ denotes the surface chloride content; *D*_0_ denotes the initial apparent chloride diffusion coefficient; *J* denotes the chloride flux; *D*_cl_ denotes the effective chloride diffuison coefficient; *C*_f_ denotes the free chloride content; *C*_t_ denotes the total chloride content; *m* denotes the aging factor; *t*_0_ denotes the curing time; *C*_smax_ denotes the maximum surface chloride content; and *r* is constant.

Most scholars have quantitatively assessed chloride ion permeability in RAC by conducting RCMT and RCPT. Some scholars have carried out indoor natural diffusion testing. A few scholars have considered chloride ion transport in unsaturated concrete. This will be discussed in detail.

### 3.2. Effect of Enhancement Methods

The implementation of enhancement methods inevitably alters the microcrack and micropore distributions in adhered mortar, ITZs, and even new mortars in RAC, which changes the channels for chloride penetration. Therefore, extensive research on chloride penetration in RAC has been conducted. In the following section, the effect of enhancement methods on the chloride resistance of RAC is reviewed.

#### 3.2.1. RA Pretreatment

##### Slurry Wrapping Treatment

In the pozzolan slurry wrapping method, fine active particles act as micro-fillers and fill the microcracks and pores of RA, followed by pozzolanic reaction at the same location [99]. The pozzolanic reaction consumes the CH generated by hydration reactions in cement and generates CSH, which helps to form denser and less permeable microstructures. Wang et al. [100] used a Vickers hardness tester to analyze the ITZs change after RA treatment. The results demonstrated that both old ITZs (ITZ_3_) and new ITZs (ITZ_2_, between old mortar and new mortar) in RAC with treated RA showed higher Vickers hardness values than those in RAC with untreated RA. Sasanipour et al. [35] compared the chloride permeability of RAC with untreated RA and SF slurry-treated RA using RCPT and RCMT. The test results showed that both the manual coating method and desiccator coating method effectively improved the chloride penetration resistance by 30–40%. The improvement effect was more remarkable for RAC with higher w/c. Kong et al. [39] developed a novel triple mixing method for coating RA with FA and fine-ground slag slurry during the concrete mixing process. Compared with normal or double mixing methods, the RAC using the developed method showed an approximately 35% lower passing charge, which was similar to that of NAC. Also, the results demonstrated that the modification effect of slag slurry was better than that of FA. In research by Wang et al. [100,101], replacing cement with a certain amount of sprayed concrete accelerator in slurry showed a better effect in decreasing the chloride migration coefficient.

Apart from volcanic ash slurry, some scholars tried to modify RA with sulphoaluminate cement (SAC) slurry [38,102]. In SAC, Ca^2+^ and Al^3+^ in minerals react with water to form calcium sulphoaluminate gels. These amorphous aluminum gels make the structure dense and durable. When coating with SAC, the sheet-like CH crystal contents in new ITZs are greatly reduced, and thus the chloride penetration resistance is improved. Using RCPT, Zhang et al. [102] demonstrated that the modification effect of RA surface coating with SAC slurry with a water-to-SAC ratio of 0.8 was 9.2%. They revealed the modification mechanism by detecting the ITZ_2_ thickness reduction and microhardness increase. In addition, with the hope of alleviating the environmental problems caused by basalt powder (BP), they also studied the modification effect of SAC–BP composite slurry. The results indicated that synergistic effect of SAC–BP was slightly better than that of SAC slurry. This was mainly due to the fact that BP filled the micropores and alleviated the excessive hydration of SAC, which may generate microcracks. To further improve the modification efficiency of SAC slurry, in another study of Zhang et al. [103] explored the effect of varying water–SAC ratios on modification efficiencies. The results showed that with a higher water–SAC ratio (1.2), the modification effect of RAC with treated RA is the best.

In addition, as sodium silicate solution can consume CH to generate CSH, water glass has been used by some researchers to precoat RA [43,103,104]. Zhang et al. [103] precoated RAs with 3 wt%, 5 wt%, and 7 wt% water glass and tested their chloride penetrations, respectively. The results indicated that 3 wt% water glass showed optimum modification efficiency (about 22%) on the chloride penetration resistance of RAC. However, water glass is not suggested for usage in concrete by some researchers as it may induce alkali–silica reactions, which form alkali–silicic acid gels, resulting in the swelling and cracking of concrete after absorbing water [105].

Currently, as nanomaterials show great potential in improving NAC performance at the nanoscale, researchers are beginning to explore the application possibilities of nanotechnology in the field of RAC [34,40,45,46,47,48]. The study results of Shaikh et al. [45] demonstrated that compared with untreated 100% RAC, the RAC with 100% RA presoaked in 2 wt% nano-silica solution for 24 h showed a 61% lower passing charge, which was even 33% lower than that of NAC. This can be attributed to the enhancement of new ITZs (ITZ_1_ and ITZ_2_) in RAC and the micro-filler effect of SiO_2_. Though the results are inspiring, it is worth mentioning that due to technical complexity, the preparation of nanomaterials is of high cost, at least for now [106,107]. Zhang et al. [46] tested and compared the chloride penetration resistance of RAC with RA presoaking in two types of slurries containing higher and lower contents of nanomaterials. The results showed that the cement–nano-silica slurry not only has a relatively controllable cost, but also exhibits an excellent modification effect on chloride penetration resistance. In addition, Li et al. [48] proposed a method of spraying SiO_2_ suspension on RA surfaces to improve the performance of RAC. However, in the study of Li et al. [34], the modification efficiency on chloride penetration was only 3.8% with the spraying method.

In summary, the modification efficiency of slurry wrapping is dependent on the slurry types, treatment methods, and concentration mix proportions. Although there has been a wealth of research results in this area, there are still many shortcomings in the current research. (i) The current research is mainly conducted through rapid testing to determine the chloride ion transport rate (RCMT, RCPT), the natural diffusion test data is still missing, and the long-term performance is still not explored. (ii) Further research is required to determine the optimal concentration of slurry. (iii) Comparative studies are still lacking in terms of both efficiency and commercial/economic considerations. Also, the effect of processing on efficiency and cost should be further considered. (iv) The current research is focused on small-scale trials, and whether large-scale application affects chloride resistance still requires further research.

##### Polymer Impregnation Treatment

In the polymer impregnation method, RA is soaked in water-soluble polymers or silane-based water-repellent polymers [41,42,43,44]. Kou and Poon [42] tested and compared the chloride penetrability of RAC with RA that was untreated or soaked in 6%, 8%, 10%, or 12% polyvinyl alcohol (PVA) solutions, respectively. They found that 10% PVA solution showed the best modification efficiency on chloride penetration resistance (35% and 32% for oven-dried and air-dried RA, respectively). This may be due to the fact that: (i) the internal pores of RA were filled with hardened polymer, which reduces porosity and pore connectivity; (ii) PVA attached to RA reduced the w/c in new ITZs (ITZ_1_ and ITZ_2_).

In summary, polymer impregnation is promising for improving the properties of RA and the chloride penetration resistance of RAC. However, there are only a few studies on the improvement of RA chloride ion transport characteristics by polymer impregnation, and these studies are mainly carried out from a macroscopic perspective. The microscopic mechanism has not yet been revealed. In addition, although Zhu et al. [108] studied the influence of silane-based water repellent either coated on the surface of the concrete or integrated into the concrete mixture on RAC’s chloride penetration, the effect of RA’s surface treatment with silane-based water-repellent polymers on chloride transport behavior still remains to be studied.

##### Carbonation Treatment

**Figure 3:**
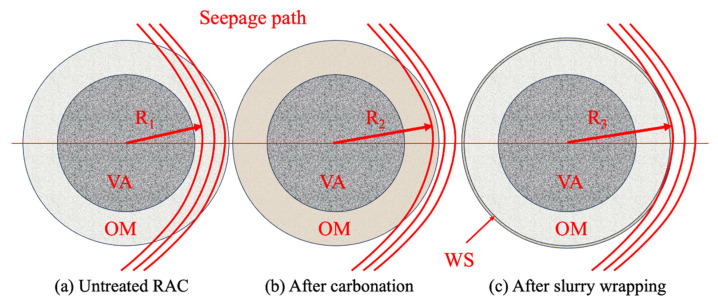
Schematics of the seepage path in (**a**) RAC; (**b**) RAC after carbonation; (**c**) RAC after slurry wrapping. VA denotes virgin aggregate.

Using the carbonation method, the densification of adhered mortar and the nucleation effect of generated calcite in new ITZs (ITZ_1_ and ITZ_2_) greatly reduced the chloride transport channels in RAC. Recently, many authors have reported the chloride penetration resistance-improving effect of RA carbonation on RAC. For example, Zhang et al. [72] tested and compared the chloride penetration resistance of untreated and RA-carbonated RAC by RCPT and natural diffusion testing, respectively. The tests indicated that compared with untreated RAC, RAC with RA which was treated under a 100% CO_2_ atmosphere at a pressure of 1 bar for 1 day showed 18.2% and 50% lower passing charges and apparent chloride diffusion coefficients, respectively. In the same case, the carbonization time was extended to 7 days and the modification effect reached 26.1% and 65.8%, indicating that a better efficiency may be achieved by accelerated carbonation. Similarly, Shi et al. [56] detected a 70.6% decrease in the chloride migration coefficient of RAC after RA accelerated carbonation curing at a 20% CO_2_ concentration for 3 days. The excellent effect may be attributed to their drying treatment at 60 °C for 48 h before RA carbonation. Additionally, they demonstrated that the enhancement efficiency of the carbonation method was slightly lower than that of SF and nano-SiO_2_ slurry wrapping treatment, but higher than that of FA slurry wrapping treatment. By a comparison test, Wang et al. [100] found that although carbonation treatment showed more efficiency in improving the microhardness of both old mortars and new ITZs compared with FA slurry wrapping treatment, the slurry wrapping method was more effective in enhancing the chloride penetration resistance. This phenomenon can be explained by a change in the transport path of chloride ions [100], as shown in Figure 3. The carbonation products clog the micropores and cracks in old mortar, resulting in a decrease in effective seepage paths and an increase in the seepage radius, while the wrapping treatment directly seals the old mortar, which makes the seepage path more tortuous, with larger seepage radius (R_3_ > R_2_ > R_1_).

**Figure 4:**
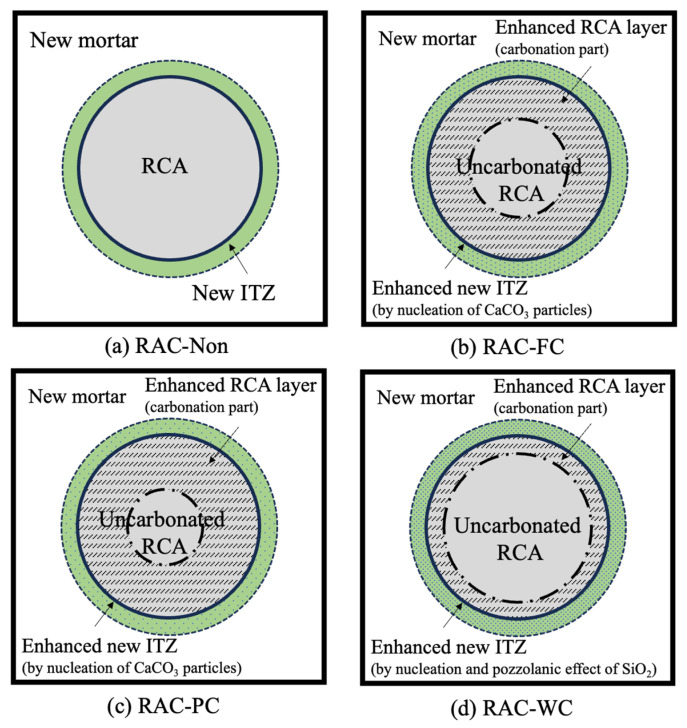

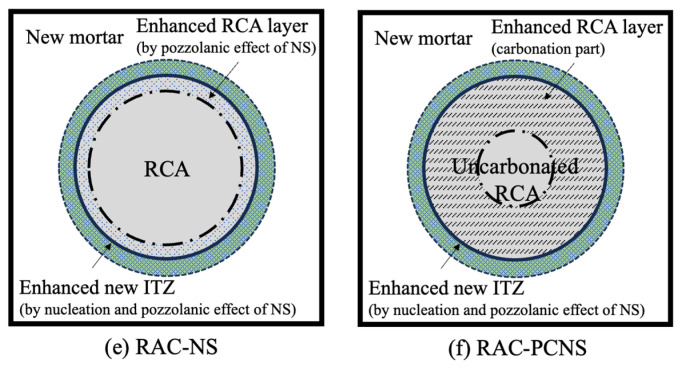
Schematics of RACs with different RCA treatment methods.

In addition, the carbonation treatment method may affect the modification efficiency. Li et al. [34] evaluated and compared the chloride penetration resistance improvement efficiency of RAC with FC, PC, and WC treatments. The chloride penetrability decreased by 11.3%, 7.4%, and 1.2%, respectively. The corresponding mechanism is described in terms of the carbonization degree of mortar and ITZ microhardness. As shown in Figure 4a–d, the order of mortar carbonization degree from largest to smallest was PC > FC > WC, while the order of new ITZ microhardness enhancement degree was WC > FC > PC [34]. Combining these two effects, the improvement effect of chloride ion resistance was FC > PC > WC. In addition, they found the modification efficiency of the PC-SiO_2_ spraying combination method was 24.4%, which was even higher than the sum of the improvement degree of the PC method and nano-SiO_2_ spraying method, respectively. This can be attributed to the synergistic effect of ITZ enhancement through the nucleation and pozzolanic effects of nano-SiO_2_ and old mortar enhancement by carbonation, as shown in Figure 4e,f.

Additionally, the study results of Xuan et al. [109] indicated that chloride penetrability decreased with the increasing carbonated RA content. Liang et al. [110] demonstrated that the chloride diffusion coefficient-decreasing percentages increased linearly with the increasing w/c of parent concrete, which indicated that the RA quality affected the modification efficiency.

It is therefore concluded that the pretreatment methods, RA quality, CO_2_ concentration, pressure, and curing time affect the chloride penetrability of RAC. Unfortunately, the effects of curing temperature and the relative humidity of CO_2_ curing on chloride penetration have not been reported until now. In addition, the data concerning chloride transport behavior under natural diffusion conditions should be further accumulated.

##### Mortar Removal Treatment

A few scholars have reported the effect of acid treatment. For example, Kim et al. [111] reported that the passing charge of RAC was decreased by 15.5% after HCl presoaking, higher than 9.8% after Na_2_SO_4_ presoaking. Also, it was found that old mortar removed by HCl presoaking was removed twice as well by Na_2_SO_4_ presoaking. Thus, the HCl treatment improved the quality of RA and chloride penetration resistance of RAC better. The study of Kazmi et al. [112] showed that the steady-state chloride migration coefficient of RAC after acetic acid treatment decreased by 10%. They also found that with rubbing treatment after acetic acid immersion, the chloride migration coefficient was further decreased by 24%. In addition, RA carbonation before acetic acid presoaking seemed to have little influence on the modification efficiency.

In summary, the type of acid affects the chloride penetration of RAC. Although some studies have proven that acid concentration can affect RA quality [113], there are no studies on the effect of acid concentration on the chloride penetration of RAC. In addition, the soaking time may affect the treatment effect, but no relevant studies have been seen.

##### Comparisons of Asserted Pre-Treated Methods

**Figure 5:**
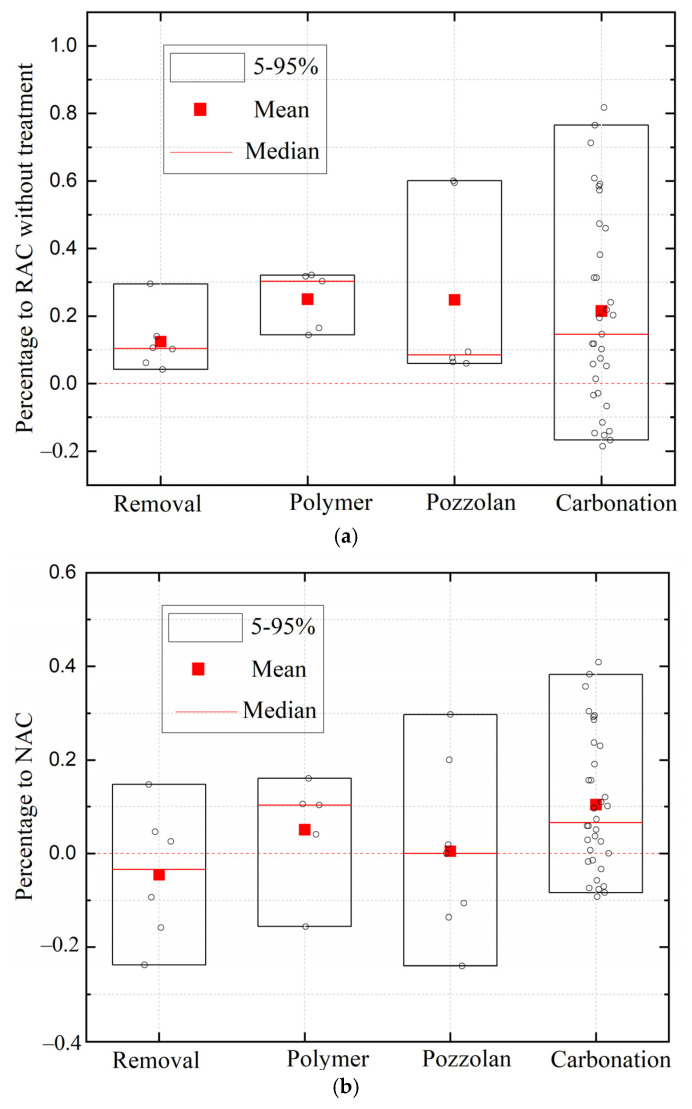
Modification efficiency in the chloride penetration resistance of RAC with mortar removal, polymer impregnation, pozzolanic slurry wrapping, and carbonation pretreatment methods: (**a**) comparison with RAC without treatment; (**b**) comparison with NAC. Adapted from Ref. [55].

Figure 5 compares the amelioration efficiency of pozzolanic slurry wrapping, polymer impregnation, carbonation, and mortar removal treatments in the chloride penetration of RAC as a whole. As shown in Figure 5, various data from the referenced literature were collected together. It is worth noting that differences in the concrete mix proportion, w/c, treatment process of RA, and experimental error contribute to the dispersion of data collected from the referenced literature. The solid rectangular box represents the 95% confidence level, the horizontal red short line represents the median, and the red dot represents the average. The red dotted line represents untreated RAC. As shown in Figure 5a, all the pre-treatment methods are effective, with a decrease in chloride penetration compared with that of RAC without treatment. The average decreases in pozzolan slurry wrapping, polymer impregnation, carbonation, and mortar removal treatments are 29%, 28%, 25%, and 15%, respectively. Also, Figure 5b presents the modification efficiency of pozzolan slurry wrapping, polymer impregnation, carbonation, and mortar removal in chloride penetration of RAC compared with NAC. It can be found that pozzolan slurry wrapping, polymer impregnation, and carbonation are promising for preparing RAC with similar or better chloride penetration resistance compared to that of NAC.

#### 3.2.2. Mineral Admixtures, Nanomaterials, or Fiber Addition

**Figure 6:**
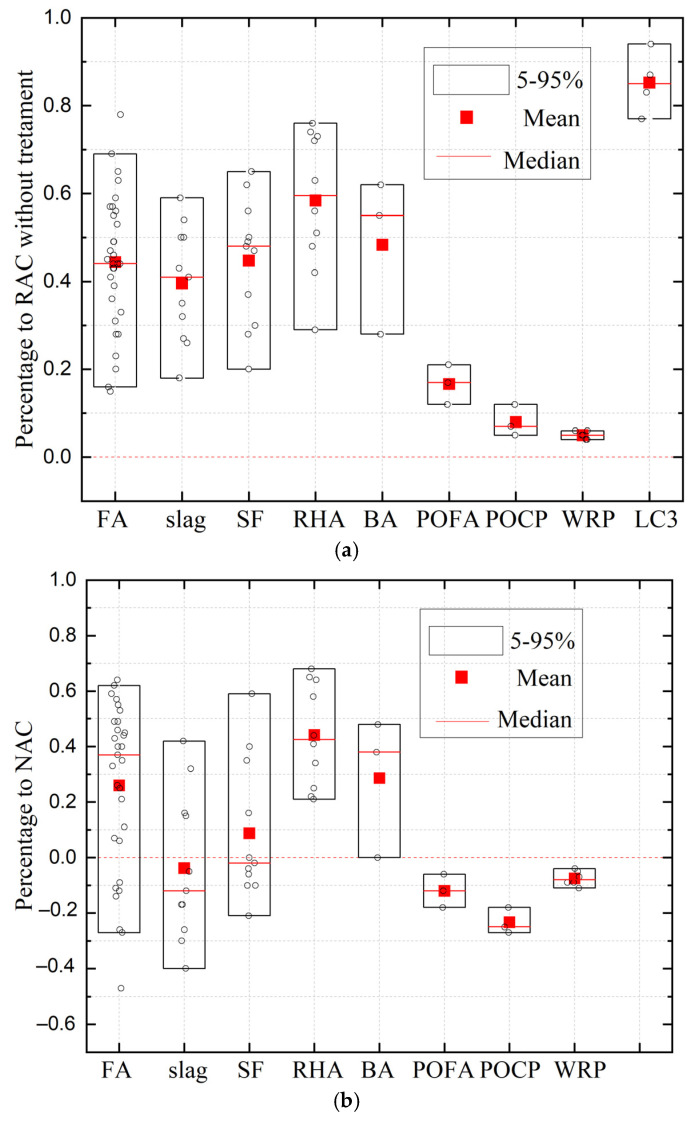
Modification efficiency in the chloride penetration resistance of RAC with assorted material additions: (**a**) comparison with RAC without treatment; (**b**) comparison with NAC. Adapted from refs. [59,110,114,115,116,117,118,119,120,121,122,124,126,127,128,129,131,132].

SCMs are a great invention for controlling the chloride ion transport properties of concrete. FA, as one of the most readily available and commonly used cement substitutes, has been widely studied for its chloride resistance. For example, the study of Sim and Park [114] demonstrated that a replacement ratio of 30% for FA to cement was able to control the chloride penetration of RAC. In addition, refs. [59,115,116,117,118,119,120,121,122] also reported the excellent performance of FA in terms of resistance to chloride ion penetration. Kou and Poon [94] found the percentage reduction in chloride ingress at 10 years of age firstly increased and then decreased with the FA content. The reason for the decrease may be that natural carbonation decreased the chloride resistance in pozzolanic mortar [123]. Kim et al. [95] concluded that the positive role of FA was not obvious at early ages, which may be due to the hydration retardation of FA. Apart from FA, slag also exhibits an efficiency effect on RAC’s chloride penetration resistance enhancement [116,118,121,124]. Kou et al. [116] compared the chloride penetration of RAC with the incorporation of different mineral admixtures. They found that FA and GGBFS only played an active role after a long curing time, while SF and metakaolin (MK) showed beneficial effects at both early and late ages. In addition, Kapoor et al. [125] demonstrated that the addition of MK was more effective in reducing chloride penetrability than equivalent SF addition. Emerging pozzolanic materials such as rice hull ash (RHA) [126,127,128], BA [129,130], palm oil fuel ash (POFA) [127], and palm oil clinker powder (POCP) [127] have also been proven to have superior effects in reducing RAC chloride ion permeability. In addition, Amiri et al. [131] tried to use waste rubber powder as replacement for cement to improve the performance, but with little success (only 4–6%). In addition to monadic binders, scholars have also studied the effects of binary or ternary SCMs. For example, as the most promising ternary alternative for traditional SCMs, limestone calcined clay cement (LC_3_) was demonstrated to reduce the chloride penetrability of RAC by 70–90% [132]. Red mud (RM) is an industrial byproduct that alone does not have the ability to improve chloride resistance, but in combination with GGBFS shows excellent performance [49]. Table 2 gives the specific data concerning the chloride diffusion coefficient/electric flux of RAC with various mineral admixtures. Figure 6a illustrates the modification efficiencies of FA, slag, SF, RHA, BA, POFA, POCP, waste rubber particles (WRP), and LC_3_ incorporation in the chloride penetration of RAC compared with RAC without treatment. From the average value comparison of statistical data, LC_3_ showed the greatest improvement efficiency in chloride ion permeability resistance of RAC (85%), followed by RAH (58%), BA, SF, FA, slag (approximately 45%), and POFA (18%). The improvement in WRP was so small that it was almost negligible (5%). Also, Figure 6b presents the modification efficiency of FA, slag, SF, RHA, BA, POFA, POCP, and waste rubber particle (WRP) incorporation in chloride penetration of RAC compared with NAC. It can be found that FA, slag, SF, RHA, and BA addition are promising for preparing RAC with similar or better chloride penetration resistance compared to that of NAC. It is worth mentioning that the amount of each admixture, water–cement ratio of concrete, and RA mass are not uniform in the collected studies. The current results can only be used as a preliminary judgment, and further tests are needed to obtain more precise results. Moreover, an apparent data dispersion can be found in Figure 6. This can be attributed to differences in the concrete design (i.e., mix proportion), concrete making environment (i.e., temperature), etc. The dispersion of some tests in Figure 6 is more obvious, which may be due to the fact that the content of mineral admixtures in different research differs. As is well known, the dosage of some admixtures shows great influence on the chloride penetration ability of concrete. Moreover, tests on POFA, POCP, WRP, and LC_3_ present obviously lower dispersion in Figure 6. This is due to the fact that current research on POFA, POCP, WRP, and LC_3_ is relatively limited. The data on such admixtures are collected from only one study, and thus show lower dispersion.

At present, nanomaterials have been studied as alternative mineral admixtures to improve the chloride ion resistance of RAC. For example, the study of Skaikh et al. [45] demonstrated that the addition of 2% nano-SiO_2_ significantly improved the chloride penetration resistance of RAC by decreasing the electric flux by 58%. A similar enhancement effect was reported in the study of Ying et al. [133]. They found the resistance of chloride penetration increased firstly and then decreased with the addition of nanomaterials (nano-SiO_2_/TiO_2_). There existed an optimum content with a 2% binder weight. The chloride migration coefficient of RAC with added nano-SiO_2_ was reduced by 33% in comparison with untreated RAC. The optimum improvement efficiency of nano-TiO_2_ was slightly higher than that of nano-SiO_2_. In addition, graphene oxide nanomaterials showed great potential in transforming RAC into durable composites [92]. However, the chloride transport behavior of RAC containing graphene oxide has still not been explored.

Apart from mineral admixtures, some studies have reported that the reinforcement of fibers in RAC augments the chloride penetration resistance. Koushkbaghi et al. [126] reported a decrease of 7% in chloride diffusivity in RAC after the addition of steel fiber. The study of Ali et al. [134] demonstrated that the chloride penetration of RAC increased with glass fiber content. However, Chen et al. [120] demonstrated that RAC’s chloride penetration increased firstly and then decreased with polypropylene fiber content. This may be because the flexible polypropylene fibers seal the voids in the mortar. Dash et al. [135] studied the effect of cellulose acetate fiber. The addition of cellulose acetate fiber had a negligible influence on the chloride penetration depth of RAC. However, at a dosage of 2.5%, the chloride penetration depth obviously increased. Zheng et al. [36] inferred that basalt fiber could effectively improve RA and ITZ. However, further studies on chloride penetrability have not been conducted.

Although scholars have conducted abundant studies on the effect of material incorporation on the chloride penetration properties of RAC, for practical application, the needed long-term natural diffusion test data, and even field test data, are still lacking.

### 3.3. Effect of the RA Replacement Ratio

**Figure 7:**
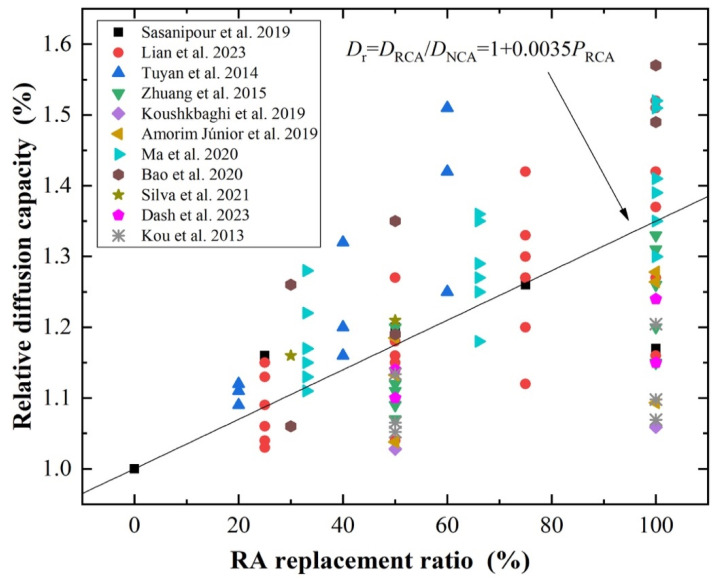
Relationship between the chloride diffusion coefficient and RA replacement ratio. Data from refs. [21,58,59,110,116,126,135,136,139,140,143,144].

The higher the RA content, the more adhered mortar and ITZs, providing more channels for chloride ingress. Many studies have shown that chloride penetration is greatly affected by the RA replacement ratio. In the study of Ma et al. [136], it was found that the chloride diffusion coefficient of RAC increased linearly with RA content. The chloride diffusion coefficient of RAC with 100% RA was found to be 51.4% higher than that of NAC. The linear increase rate of the diffusion coefficient with RA content is more obvious at earlier ages. Through RCM conducted according to GB/T 50082-2009 [137] and NT Build 492 [138], as reported in [58,139], the chloride migration coefficient of RAC at 28 d of curing was obtained. It was found that the increasing degrees of the chloride migration coefficient in RAC at w/c values of 0.45, 0.39, and 0.33 were 57%, 48%, and 27%, respectively. This indicated that the sensitivity of RA content to chloride penetration is more remarkable in RAC with higher w/c values. Sasanipour et al. [110], Tuyan et al. [140], and Kou et al. [59,116] conducted RCPT according to ASTM C1202 [141] to obtain the passing charges of RAC with different RA contents. The results also demonstrated a linear relationship between the diffusion coefficient and RA content (the chloride diffusion coefficient was obtained by conversion of passing charges [142]). Through wetting–drying cyclic tests, similar findings were obtained in the study of Lian et al. [21] and Dash et al. [135]. According to refs. [21,58,59,110,116,126,135,136,139,140,143,144], the relationship between the ratio of *D*_RAC_ to *D*_NAC_ (*D*_r_) and the substitution ratio of RA was obtained statistically (Figure 7). It can be found that the relationship between *D*_r_ and RA content is basically linear. For RAC with 100%, *D*_r_ ranged between 10 and 60% [126,139]. This may be due to the fact that w/c and RA qualities and types also affect the relationship between RAC diffusivity and RA content [54].

**Figure 8:**
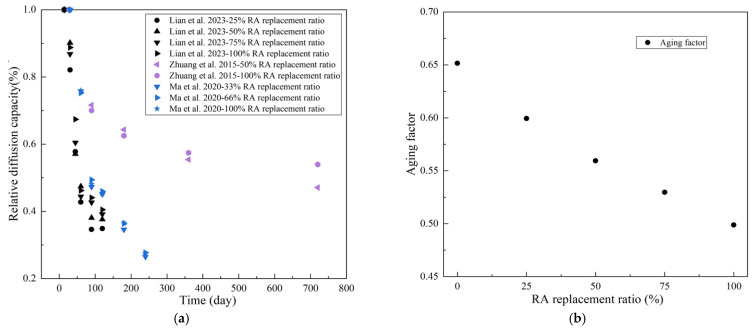
(**a**) Time-dependent relative chloride diffusion coefficient; (**b**) aging factor. Data from refs. [39,116,123].

In addition, RA content also affects the time-varying characteristic of *D* in RAC. Figure 8a illustrates the changes in relative diffusion capacity (ratio of the real-time diffusion coefficient to the initial diffusion coefficient) over time [21,58,136]. It can be found that diffusion coefficient of concrete decreases as the hydration time increases. This is due to the pore refinement in concrete owing to hydration reactions. In addition, the decrease degree of *D* was found to be inversely proportional to RA content at the same age. Figure 8b provides the relationship between RA content and the aging factor, which characterizes the evolution rate of *D* as per Equation (3) [21]. It can be clearly found that *m* decreases nearly linearly with the increase in RA content. This indicates that RA to some extent hinders variation in porosity.

**Figure 9:**
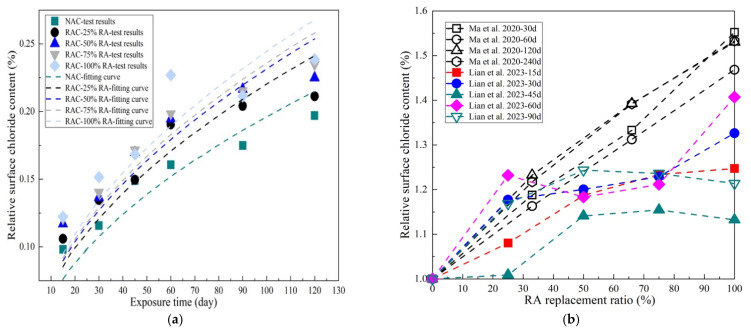
(**a**) Time-dependent relative surface chloride content; (**b**) the relationship between relative surface chloride content and the RA replacement ratio. Data from refs. [21,136].

As a comprehensive result of concrete properties and environmental impact, the surface chloride content is another crucial parameter to describe the chloride transport behavior in RAC [21,136]. As shown in Figure 9a, *C*_s_ increases over time, obeying the exponential form or power function form [97]. The higher the RA content, the higher the *C*_s_ at the same age. Ma et al. [136] reported a similar finding. They found that the *C*_s_ of RAC increases linearly with RA content. This may be due to the increase in surface porosity caused by RA leading to more chloride ion precipitation. In addition, Lian et al. [21] found that the increase rate of *C*_s_ increases with RA content.

### 3.4. Effect of RA Quality

In practice, RAs are obtained from different demolished structures and thus pose distinctive quality. Numerous studies have shown that RA quality has certain effects on the content and porosity of old mortar, thus affecting the chloride transport behavior in RAC. Knowledge of the impact of RA quality on chloride transport behavior in RAC plays an important role for infrastructure stakeholders to control the quality of RAC structures in practical applications.

Pedro et al. [145] compared the chloride penetration of RAC with RA sourced from the laboratory and pre-casting industry, respectively. They found that RAC from different sources showed a difference of 20% in its chloride diffusion coefficient. This may be due to the fact that chloride resistance is mainly sensitive to the parent concrete strength, which mainly affects the quality of adhered mortar in RA. Moreover, they found that the performance of RAC with RA obtained by two distinct crushing processes showed negligible differences. However, different crushers do affect the quality of RA [146]. Further study on the effect of crushing methods on chloride penetration resistance is needed. The study of Bao et al. [139] showed that the RA types had a remarkable influence on the chloride penetration of RAC since the water absorption of different RAs was distinctive. Kou et al. [147] detected the chloride penetrability of RAC with RA derived from 30 MPa to 100 MPa parent concrete. The passing charge was found to decrease with the increasing parent concrete strength, and RAC produced by RA derived from 100 MPa parent concrete showed comparable chloride resistance to NAC. They concluded that RA derived from 80–100 MPa concrete can totally replace NA for high-performance concrete production. A similar relationship between the chloride penetration resistance of RAC and parent concrete strength was reported in ref. [148]. This is due to the fact that, although the stronger bond between mortar and aggregate in stronger concrete caused higher old mortar content in RA, the RA quality was better in stronger parent concrete with less porous mortar [149]. Specifically, the water absorption of RA derived from stronger parent concrete decreased [147]. Thus, the generated RAC showed a lower absorption and volume of voids [148]. However, paradoxical discoveries were reported by Gholampour et al. [150], Duan et al. [151], and Chakradhara Rao et al. [152], who found that the water absorption of RA increased with the increase in parent concrete strength. Although they did not report the influence on chloride resistance, further study is needed to analyze the possible reason and mechanism or to provide a comprehensive analysis taking all possible influence factors into consideration since the chloride resistance of RAC has been demonstrated to be linear with RA water absorption [54].

Multi-recycling of concrete can further save resources, protect the environment, and reduce project construction costs [153]. The number of cycles is also an influential factor in RA quality. It has been demonstrated that the chloride penetration resistance decreases with increasing generations of RA [143,153]. This due to the accompanying higher old mortar content with the increase in the cycle number. Furthermore, the micro-pores in RA were found to be increased by SEM morphology analysis, which is due to the spherical vaterite being transformed into rhombohedral calcite. When the number of recycling cycles was larger, the chloride resistance showed more sensitivity to the RA replacement ratio [143]. The current study mainly focused on RA with no more than three generations. Although an asymptotic behavior was detected, three generations is not enough to determine the stable properties of RAC [143].

RA of different quality is mainly reflected in different water absorption values, crushing values, binder mortar contents, and porosities. Kazmi et al. [112] claimed that these physical properties of RA directly affect the durability of RAC. Based on data from the literature, they established regression equations for predicting the chloride migration coefficient from asserted RA properties, respectively, as shown in Table 3. Similarly, based on data from the literature, Liang et al. [54] established the regression equations between the relative chloride diffusion coefficient and the average rate of water absorption.

Although the research on the relationship between chlorine resistance and RA quality has achieved abundant results, only some preliminary attempts have been made to examine chloride transport behavior so far [143,153,154]. Current studies mainly focus on the measurement of the RAC durability index, and a database involving long-term chloride ion transport behavior should be established by further research.

### 3.5. Effect of External Environment Damage

#### 3.5.1. Effect of Freeze–Thaw Cycles

**Figure 10:**
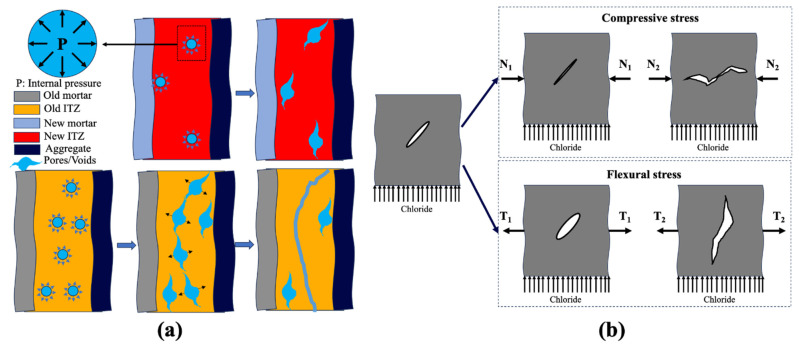
Schematics of (**a**) freeze–thaw damage mechanisms of NAC and RAC; (**b**) the cracking development process of RAC suffering compressive and flexural stress.

Under the action of freeze–thaw cycles, the water in concrete voids repeatedly causes ice expansion pressure and osmotic pressure, thus causing damage inside the concrete by forming fatigue stress. When the damage gradually accumulates and expands, it develops into interconnected cracks, which gradually reduce the strength of concrete. As proposed by Júnior et al. [155], freeze–thaw cycles are more damaging to RAC than NAC. In comparison with NAC, the old mortar and ITZs in RA contain more voids and microcracks, which are more likely to develop into interconnecting cracks (as shown in Figure 10a); moreover, the presence of more heterogeneous interfaces further threatens the freeze–thaw resistance of RAC [156]. Ma et al. [136] studied the chloride permeability of RAC after pre-damage by freeze–thaw cycles using chloride diffusivity testing. They found that the freeze–thaw damage of RAC increased with the RA percentage during the same freeze–thaw cycles. In addition, the chloride diffusivity was found to increase linearly with pre-damage characterized by a relatively dynamic elastic modulus, and the damage accumulation rate increased with the increase in RA percentage, which means that the existence of RA increased the sensitivity of chloride diffusivity to freeze–thaw damages. In the actual working environment, chloride ion erosion is accompanied by freeze–thaw cycles. Hao et al. [157] studied the chloride diffusivity of RAC subjected to salty freeze–thaw cycles. They found the chloride concentration increased with RA content during the same freeze–thaw cycles. Although the current research has yielded some results, the effect on the chloride diffusion coefficient and surface chloride content has not been reported, which is necessary for describing chloride ingress behavior.

#### 3.5.2. Effect of External Loads

In comparison with NAC, there are many defects in RAC. Thus, it is expected that RAC is more sensitive to external loads. Ma et al. [136] determined the damage level of RAC under a compressive load using an ultrasonic concrete tester, and then established the relationship between the key parameters describing chloride transport behavior and damage levels. They found both surface chloride content and chloride diffusion coefficient linearly increased with the damage level, and the higher the replacement ratio of RA, the more sensitive these parameters were to load damage. Similar findings were reported in the study of Peng et al. [158]. Wang et al. [159] established a quantitative relationship between the compressive load stress ratio and the diffusion coefficient. The results indicated that a critical stress ratio around 0.40–0.60 existed. Below this threshold, the diffusion coefficient decreases linearly with the stress ratio, and vice versa. Wang et al. [160] reported similar findings. The difference is that in their results, the threshold was approximately 0.2. This may be due to differences in test methods. The former test applied load damage to the specimen first and then measured the diffusion coefficient, while the latter measured the coefficient under a continuous load. In addition, Wang et al. [160] studied the chloride penetration under flexural stress. A linear, increasing relationship was found between the flexural stress ratio and the chloride diffusion coefficient. They also offered an explanation. As shown in Figure 10b, the different forms of voids-to-cracks development under compressive loading and tensile loading result in different chloride ion transport characteristics.

### 3.6. Binding Capacity of RAC

When free chloride ions enter the concrete from the external environment, some of them still exist in the pore solution in a free-state form, while some of them are physically or chemically adsorbed and exist a bound-state form [161]. It is the free chloride ions that cause the depassivation of the reinforcement rather than the binding chloride ions [162]. On one hand, the binding of chloride decreased the free chloride content in concrete. On the other hand, the binding phenomenon increased the chloride threshold (which is expressed as the total chloride content). Therefore, some studies researched the chloride binding capacity of RAC. Xiao et al. [163] found a linear relationship between binding and free chloride content in both RAC and NAC. Zaccardi et al. [20] found the chloride binding capacity was higher in RAC compared with NAC. In the study of Lian et al. [21], the chloride binding capacity increased linearly with the RA content in RAC. This may be due to the old mortar in RAC containing calcium sulfoaluminate (AFm) and CSH. The former reacted with chloride to form Friedel’s salt, while the latter physically bound chloride ions. In addition, chloride ion-binding capacity increases with w/c, and effective w/c is generally higher due to the higher water absorption of RA. Similar findings were reported in the study of Qi et al. [118]. However, Bai et al. [164] reported conflicting research results. They found that the addition of RA decreased the chloride binding capacity in concrete. Due to the existence of contradictory results, further research is needed.

## 4. Chloride Ingress-Based Service Life Prediction

Chloride ingress-based service life prediction denotes quantitative estimates of the time-dependent state of reinforced concrete structures based on the deterioration induced by chloride transport. As proposed by Alexander et al. [165], service life prediction helps to select combination designs of concrete covers and concrete material properties, which is especially necessary before the application of modern materials, such as RAC. Service life prediction includes chloride ingress modeling and limit state modeling. These models can be deterministic or probabilistic. Some studies have reported on mathematical, analytical, numerical, and empirical service life models of RAC, and some of them have even been based on artificial neural networks [144,165,166,167].

For example, Chen et al. [166] used a deterministic model to predict the service lives of RAC with different RA replacement ratios, polypropylene fiber contents, and water reducing and air entraining agent mix proportions based on the analytical solution of Fick’s Second Law. The input parameter chloride diffusion coefficient was determined by chloride conductivity testing according to the China Civil Engineering Society standard (CCES2004) [168]. They also considered the influence of microcracks by introuducing the influence factor *k*. Similarly, the surface chloride content and chloride threshold were determined by empirical formulas. In this way, they found the service life of RAC decreased with the increasing RA replacement ratio, and the addition of polypropylene fiber had a negligible impact, while the service lives of RAC increased firstly with the mix proportion of water reducing and air entraining agent and peaked at 0.4%. In a similar way, Júnior et al. [144] estimated the service life of RAC with different RA contents. The difference is that they determined the non-steady-state diffusion coefficient by combining chloride migration testing and electrical resistivity testing. Details of the relevant test methods can be found in ref. [169]. They found an order reduction of 40% to 55% in service life with the total substitution of NCA for RCA.

Although deterministic modeling provides a simple method to compare the service lives of concrete structures, some researchers [168,170] have claimed that probabilistic modeling takes statistical variability into account. Ying et al. [171] developed and verified a probability density evolution method to predict the probability distribution of the chloride diffusion coefficient of RAC, *D*_RAC_. They claimed that the probability distribution of *D*_RAC_ can be obtained by the probability distribution of the content and chloride diffusion coefficient of old mortar. Albuquerque et al. [170] calibrated the concrete cover of RAC according to Eurocode Design using the probabilistic depassivation model as per Fib Bulletin 34 [172]. For stochastic modeling of the chloride migration coefficient, they conducted RCMT with 13–15 samples for each mix proportion to determine the standard deviation and mean value. It was found that the variability in RAC’s chloride migration coefficient was smaller than that of NAC. By comparing the reliability index of RAC and NAC, they recommended an increase in concrete cover of 5 mm for RAC to achieve a similar reliability index to that of NAC. However, apart from *D*_RCM_, the mean values of other key parameters like the ageing exponent, surface chloride content, and critical chloride content were determined using an empirical formula established based on NAC. In the probabilistic model of Wang et al. [27], the mean values of these parameters were determined by laboratory tests. Moreover, they studied the reinforcement corrosion rate in RAC, based on which the service life with concrete cracking as the limit state was determined. It was found that the use of SCMs significantly improved the service life of RAC, which paved the path for the application of RAC in marine engineering.

Apart from the aforementioned models, Stambaugh et al. [173] implemented and verified a 1D numerical service-life prediction model for reinforced RAC. They solved Fick’s Second Law using the finite difference method. Based on the empirical formulas and probabilistic models of input parameters, they found that the w/c ratio and aggregate replacement ratio exhibited the greatest impact on service life with the limit state of concrete cracking.

In summary, abundant results have been achieved concerning the service life modeling of RAC structures, which provides confidence for the design and application of RAC structures in chloride-ingressive environments. However, there are still many limitations in current research. (i) At present, most of the key parameters for the service life modeling of RAC structures are determined based on the empirical values of NAC structure practices. Some used the data collected from laboratory test results. However, the current laboratory tests usually provide short-term data. Therefore, the reliability of short-term laboratory tests to predict the long-term performance of RAC needs to be verified through long-term real marine exposure test data, and there is always a substantial difference between the conditions of the laboratory and the conditions of real marine environments. Therefore, it is of great scientific importance to carry out real marine exposure tests and compare and analyze the results of laboratory testing and long-term exposure testing to select material schemes with excellent durability and accurately evaluate the design service life of offshore structures. (ii) At present, prescriptive provisions for controlling the durability of RAC structures are still lacking. It is an inevitable trend to formulate appropriate provisions, thus making it convenient for designers and engineers to control the durability deterioration of RAC structures. (iii) Current studies on service life modeling mainly take reinforcement initiation corrosion or concrete cracking as limit states. Suitable and reliable models for corrosion propagation, e.g., spalling, collapse of the structure, etc., are still missing.

## 5. Conclusions and Outlook

### 5.1. Conclusions

This paper presents a review of the current research on the chloride penetration properties of RAC with enhancement treatment and systematically summarizes (i) the RAC property enhancement methods; (ii) the effect of enhancement methods, the RA replacement ratio, RA quality, and external environmental damages on the chloride transport properties of RAC; (iii) chloride-binding capacity in RAC with enhancement treatment; and (iv) chloride-ingress-based service life prediction for enhanced RAC structures. The chloride penetration resistance modification efficiency of enhancement methods is discussed and compared. From the above analysis, it can be seen that both old mortar modification and removal methods effectively improve the chloride penetration resistance of RAC. The chloride penetration of RAC is strongly correlated with the RA replacement ratio and RA quality. The chloride penetration of concrete containing RA shows higher sensitivity to freeze–thaw damage and external loads compared with NAC. Enhanced RA treatment is beneficial to reduce this sensitivity. In addition, the chloride-ingress-based service life prediction of RAC demonstrates that enhancement treatment obviously improves the estimated service life of RAC structures.

### 5.2. Recommendations for Further Research

(1) Even though some studies have reported on modification efficiency in chloride penetration resistance and the time-dependent chloride transport behavior of RAC with enhancement treatment, the relationship between specific treatment conditions and RAC’s chloride penetration resistance is still not clear. The influence of treatment conditions on modification efficiency needs to be studied, and optimal treatment conditions should be determined.
(2) At present, some studies have examined the influence of RA quality, types, and parent concrete types on the chloride penetration resistance of modified RAC, which is mainly characterized by the chloride diffusion coefficient *D*_RCM_, etc. However, the influence of the above factors on the time-dependent chloride transport behavior of RAC is still not clear.
(3) Although current studies have shown the influence of external environment damage (i.e., freeze–thaw, external loads) on the chloride diffusivity of modified RAC, proper modeling to describe the time-dependent chloride transport behavior of RAC structures exposed to real marine freeze–thaw environments or external loads in different forms should be further established.
(4) Current research has studied the chloride binding behavior of RAC. However, there is little research on the influence of enhancement treatment on the chloride binding capacity of RAC.
(5) Furthermore, a durability design method for RAC structures with enhancement treatments in marine environments should be developed.

## Figures and Tables

**Table 1:** Slurry wrapping treatment/polymer impregnation treatment for the enhancement of RA.

Implementation Measures	Mechanism	Improvement Effect	
		WA Reduction	CS Increase
Silica fume (SF) slurry immersion (at 8% cement weight) [35]	Introducing active particles and filling pores.	14–23%	1%
Portland cement slurry [38]	Filling effect.	39%	1%
Fly ash (FA) slurry [56]	Reacting with CH to form CSH.	24.5%	21%
Pozzolana slurry (SF + FA) [63]	Forming CSH, filling the cavities.	45%	17.8%
SF slurry [56]	Reacting with CH to form CSH.	20.7%	60.5%
Nano-SiO_2_ slurry [56]	Reacting with CH to form CSH.	20.7%	42%
Nano-silica spraying [34]	Reacting with CH to form CSH.	3.7%	5.7%
Calcium metasilicate solution [64]	Forming a protective coating.	2.7–3.6%	8%
Diammonium hydrogen phosphate solution [60]	Precipitating hydroxyapatite.	18.1%	18.9%
Sodium silicate solution [43]	Water-repellent post-treatment.	55%	/
PVA modification (oven-drying) [42]	Improving surface activity and workability.	61.6%	1%
PVA modification (air-drying) [42]		74%	0%
Silane solution [65]	Surface water-repellent treatment.	46%	/
Paraffin wax [65]	Surface water-repellent treatment.	83%	/
Octyl/methyl methoxy co-oligomeric siloxane/silane [43]	Water-repellent post-treatment.	56%	/
Octyl triethoxy silane [43]	Water-repellent post-treatment.	86%	/
Siloxane/propyl trimethoxy silane [43]	Water-repellent post-treatment.	73%	/
Siloxane/propyl triethoxy silane [43]	Water-repellent post-treatment.	55%	/
Siloxane/alkyalkoxysilane [43]	Water-repellent post-treatment.	53%	/
Water glass [66]	Creating a smooth, dense, and hard coating.	67–77%	9%

WA—water absorption; CS—compressive strength; CH—calcium hydroxide. CSH—calcium silicate hydrate; PVA—polyvinyl alcohol.

**Table 2:** Chloride diffusion coefficient/electric flux of RAC with various mineral admixtures.

Added Materials	w/c	RA Content	Curing Age	Addition Content	Chloride Diffusion Coefficient (D = 10^−12^ m^2^/s)/Electric Flux (C)	Efficiency	Reference
FA	0.45	100%	180 d	30%	3522 C	47%	[117]
FA	0.35	100%	56 d	15%	294 C	44%	[114]
FA	0.35	100%	56 d	30%	164 C	69%	[114]
FA	0.43	100%	91 d	30%	4.04 D	78%	[115]
FA	0.5	100%	270 d	30%	7.53 D	16%	[118]
FA	0.5	100%	90 d	35%	2916 C	28%	[116]
FA	0.53	100%	28 d	30%	8.5 D	39%	[119]
FA	0.53	100%	90 d	30%	4.7 D	57%	[119]
FA	0.53	100%	180 d	30%	3.63 D	59%	[119]
FA	0.53	100%	365 d	30%	3 D	65%	[119]
FA	0.53	100%	28 d	60%	11.9 D	15%	[119]
FA	0.53	100%	90 d	60%	5.95 D	45%	[119]
FA	0.53	100%	180 d	60%	4.1 D	53%	[119]
FA	0.53	100%	365 d	60%	3.2 D	63%	[119]
FA	0.4	40%	28 d	45%	1.23 D	36%	[120]
FA	0.4	70%	28 d	45%	1.28 D	31%	[120]
FA	0.35	100%	28 d	25%	3.87 D	23%	[121]
FA	0.4	100%	28 d	25%	4.5 D	20%	[121]
FA	0.45	100%	28 d	25%	5.42 D	44%	[121]
FA	0.5	100%	28 d	25%	6.03 D	43%	[121]
FA	0.55	100%	28 d	25%	6.88 D	55%	[121]
FA	0.6	100%	28 d	25%	8.61 D	56%	[121]
FA	0.45	100%	28 d	30%	4226 C	/	[122]
FA	0.45	100%	56 d	30%	2403 C	/	[122]
FA	0.45	100%	120 d	30%	931 C	/	[122]
FA	0.55	100%	28 d	25%	4977 C	28%	[59]
FA	0.55	100%	1 year	35%	4634 C	33%	[59]
FA	0.55	100%	10 years	55%	3959 C	43%	[59]
FA	0.55	100%	28 d	25%	3005 C	41%	[59]
FA	0.55	100%	1 year	35%	2598 C	49%	[59]
FA	0.55	100%	10 years	55%	2208 C	57%	[59]
FA	0.55	100%	28 d	25%	2185 C	44%	[59]
FA	0.55	100%	1 year	35%	2078 C	46%	[59]
FA	0.55	100%	10 years	55%	1987 C	49%	[59]
Slag	0.45	100%	180 d	65%	3299 C	50%	[59]
Slag	0.4	100%	1 year	50%	1.11 D	50%	[124]
Slag	0.4	100%	1 year	70%	1.3 D	41%	[124]
Slag	0.5	100%	270 d	30%	7.12 D	26%	[118]
Slag	0.5	100%	90 d	55%	2750 C	32%	[116]
Slag	0.35	100%	28 d	25%	3.69 D	27%	[121]
Slag	0.4	100%	28 d	25%	4.61 D	18%	[121]
Slag	0.45	100%	28 d	25%	5.55 D	43%	[121]
Slag	0.5	100%	28 d	25%	6.78 D	35%	[121]
Slag	0.55	100%	28 d	25%	7.08 D	54%	[121]
Slag	0.6	100%	28 d	25%	7.99 D	59%	[121]
SF	0.5	100%	90 d	10%	3267 C	20%	[116]
SF	0.35	100%	28 d	25%	3.17 D	37%	[121]
SF	0.4	100%	28 d	25%	3.92 D	30%	[121]
SF	0.45	100%	28 d	25%	4.97 D	49%	[121]
SF	0.5	100%	28 d	25%	5.52 D	47%	[121]
SF	0.55	100%	28 d	25%	6.7 D	56%	[121]
SF	0.6	100%	28 d	25%	7.54 D	62%	[121]
SF	0.4	25%	28 d	8%	1455 C	65%	[110]
SF	0.4	50%	28 d	8%	2152 C	50%	[110]
SF	0.4	75%	28 d	8%	2330 C	48%	[110]
SF	0.4	100%	28 d	8%	2989 C	28%	[110]
MK	0.5	100%	90 d	15%	3175 C	22%	[116]
RHA	0.55	100%	90 d	10%	2078 C	51%	[127]
RHA	0.55	100%	90 d	20%	991 C	76%	[127]
RHA	0.55	100%	90 d	30%	1086 C	74%	[127]
RHA	0.45	100%	90 d	20%	17.8 D	48%	[128]
RHA	0.45	100%	90 d	35%	12.8 D	63%	[128]
RHA	0.45	100%	90 d	50%	9.6 D	72%	[128]
RHA	0.45	100%	180 d	20%	13.9 D	42%	[128]
RHA	0.45	100%	180 d	35%	10.4 D	56%	[128]
RHA	0.45	100%	180 d	50%	6.4 D	73%	[128]
RHA	0.35	100%	90 d	14%	5.2 D	29%	[126]
BA	0.3	100%	90 d	20%	5 D	28%	[129]
BA	0.3	100%	90 d	35%	3.13 D	55%	[129]
BA	0.3	100%	90 d	50%	2.61 D	62%	[129]
POFA	0.55	100%	90 d	10%	3301 C	21%	[127]
POFA	0.55	100%	90 d	20%	3491 C	17%	[127]
POFA	0.55	100%	90 d	30%	3695 C	12%	[127]
POCP	0.55	100%	90 d	10%	3899 C	7%	[127]
POCP	0.55	100%	90 d	20%	3682 C	12%	[127]
POCP	0.55	100%	90 d	30%	3980 C	5%	[127]
WRP	0.35	50%	90 d	2.50%	10.2 D	4%	[131]
WRP	0.35	50%	90 d	5%	10 D	6%	[131]
WRP	0.4	50%	90 d	2.50%	10.1 D	6%	[131]
WRP	0.4	50%	90 d	5%	10.2 D	5%	[131]
WRP	0.45	50%	90 d	2.50%	10.1 D	4%	[131]
WRP	0.45	50%	90 d	5%	10 D	5%	[131]
LC_3_	0.4	100%	28 d	30%	3.23 D	77%	[132]
LC_3_	0.4	100%	28 d	50%	2.42 D	83%	[132]
LC_3_	0.4	100%	300 d	30%	1.31 D	87%	[133]
LC_3_	0.4	100%	300 d	50%	0.57 D	94%	[132]
FA-SF	0.45	100%	28 d	30%/10%	1844 C	/	[122]
FA-SF	0.45	100%	56 d	30%/10%	776 C	/	[122]
FA-SF	0.45	100%	120 d	30%/10%	691 C	/	[122]
RM-slag	0.5	100%	90 d	10–15%	2.85 D	18%	[49]
RM-slag	0.5	100%	90 d	10–35%	1.94 D	44%	[49]
RM-slag-GP	0.5	100%	90 d	7.5–10–7.5%	3.12 D	10%	[49]
RM-slag-GP	0.5	100%	90 d	5–35–5%	2.29 D	34%	[49]
Steel Fiber	0.35	100%	90 d	76 kg/m^3^	6.8 D	7%	[126]
RHA-Steel fiber	0.35	100%	90 d	14%/76 kg/m^3^	4.6 D	37%	[126]

**Table 3:** Relationships between the chloride migration coefficient and RA properties.

Outcome Variable	Predictor	Model	Coefficient of Determination
Chloride migration coefficient (*D*_RCM_) (×10^−12^ m^2^/s)	Water absorption (*WA*) (%)	*D*_RCM_ = 0.75 *WA* + 18.97	0.32
	Crushing value (*CV*) (%)	*D*_RCM_ = 6.00 e^0.05CV^	0.32
	Old mortal content (*OMC*) (%)	*D*_RCM_ = 20.33 e^0.004OMC^	0.26
	Porosity (*P*) (%)	*D*_RCM_ = 0.35 *P* + 18.26	0.52

## References

[1] Wang D., Lu C., Zhu Z., Zhang Z., Liu S., Ji Y., Xing Z. (2023). Mechanical Performance of Recycled Aggregate Concrete in Green Civil Engineering: Review. Case Stud. Constr. Mater..

[2] Oikonomou N.D. (2005). Recycled Concrete Aggregates. Cem. Concr. Compos..

[3] Maaze M.R., Shrivastava S. (2023). Design Optimization of a Recycled Concrete Waste-Based Brick through Alkali Activation Using Box-Behnken Design Methodology. J. Build. Eng..

[4] European Commission (DG ENV) (2011). Report on the Management of Construction and Demolition Waste in the EU. ARCADIS Infrastructure Environment Facilities. https://environment.ec.europa.eu/document/download/22239ead-82d4-42fb-86dc-d202d5f40507_en?filename=2011_CDW_Report_0.pdf.

[5] Munir M.J., Kazmi S.M.S., Wu Y.-F., Patnaikuni I., Zhou Y., Xing F. (2020). Stress strain performance of steel spiral confined recycled aggregate concrete. Cem. Concr. Compos..

[6] Poon C.S., Shui Z.H., Lam L. (2002). Strength of Concretes Prepared with Natural and Recycled Aggregates at Different Moisture Conditions. Advances in Building Technology.

[7] Poon C.S., Shui Z.H., Lam L. (2004). Effect of Microstructure of ITZ on Compressive Strength of Concrete Prepared with Recycled Aggregates. Constr. Build. Mater..

[8] Ridzuan A.R.M., Diah A.B.M., Hamir R., Kamarulzaman K.B. (2001). The Influence of Recycled Aggregate on the Early Compressive Strength and Drying Shrinkage of Concrete. Structural Engineering, Mechanics and Computation.

[9] Ajdukiewicz A., Kliszczewicz A. (2002). Influence of Recycled Aggregates on Mechanical Properties of HS/HPC. Cem. Concr. Compos..

[10] Akita H., Koide H., Ojima M. (2003). Tensile behavior of a recycled concrete. Brittle Matrix Composites 7.

[11] Tabsh S.W., Abdelfatah A.S. (2009). Influence of Recycled Concrete Aggregates on Strength Properties of Concrete. Constr. Build. Mater..

[12] Olorunsogo F.T., Padayachee N. (2002). Performance of Recycled Aggregate Concrete Monitored by Durability Indexes. Cem. Concr. Res..

[13] Thomas C., Setién J., Polanco J.A., Alaejos P., Sánchez De Juan M. (2013). Durability of Recycled Aggregate Concrete. Constr. Build. Mater..

[14] Levy S.M., Helene P. (2004). Durability of Recycled Aggregates Concrete: A Safe Way to Sustainable Development. Cem. Concr. Res..

[15] Henry M., Pardo G., Nishimura T., Kato Y. (2011). Balancing Durability and Environmental Impact in Concrete Combining Low-Grade Recycled Aggregates and Mineral Admixtures. Resour. Conserv. Recycl..

[16] Abbas A., Fathifazl G., Isgor O.B., Razaqpur A.G., Fournier B., Foo S. (2009). Durability of Recycled Aggregate Concrete Designed with Equivalent Mortar Volume Method. Cem. Concr. Compos..

[17] Richardson A., Coventry K., Bacon J. (2011). Freeze/Thaw Durability of Concrete with Recycled Demolition Aggregate Compared to Virgin Aggregate Concrete. J. Clean. Prod..

[18] Bravo M., De Brito J., Pontes J., Evangelista L. (2015). Durability Performance of Concrete with Recycled Aggregates from Construction and Demolition Waste Plants. Constr. Build. Mater..

[19] Debieb F., Courard L., Kenai S., Degeimbre R. (2010). Mechanical and Durability Properties of Concrete Using Contaminated Recycled Aggregates. Cem. Concr. Compos..

[20] Villagrán-Zaccardi Y.A., Zega C.J., Di Maio Á.A. (2008). Chloride Penetration and Binding in Recycled Concrete. J. Mater. Civ. Eng..

[21] Lian S., Meng T., Zhao Y., Liu Z., Zhou X., Ruan S. (2023). Experimental and Theoretical Analyses of Chloride Transport in Recycled Concrete Subjected to a Cyclic Drying-Wetting Environment. Structures.

[22] Srubar W.V. (2015). Stochastic Service-Life Modeling of Chloride-Induced Corrosion in Recycled-Aggregate Concrete. Cem. Concr. Compos..

[23] Letelier V., Hott F., Bustamante M., Wenzel B. (2024). Effect of recycled coarse aggregate treated with recycled binder paste coating and accelerated carbonation on mechanical and physical properties of concrete. J. Build. Eng..

[24] Kim J. (2022). Influence of Quality of Recycled Aggregates on the Mechanical Properties of Recycled Aggregate Concretes: An Overview. Constr. Build. Mater..

[25] Zheng Y., Xi X., Zhang Y., Zhang P., Du C. (2023). Review of Mechanical Properties and Strengthening Mechanism of Fully Recycled Aggregate Concrete under High Temperature. Constr. Build. Mater..

[26] Deresa S.T., Xu J., Demartino C., Heo Y., Li Z., Xiao Y. (2020). A Review of Experimental Results on Structural Performance of Reinforced Recycled Aggregate Concrete Beams and Columns. Adv. Struct. Eng..

[27] Bahraq A.A., Jose J., Shameem M., Maslehuddin M. (2022). A review on treatment techniques to improve the durability of recycled aggregate concrete: Enhancement mechanisms, performance and cost analysis. J. Build. Eng..

[28] Shahjalal M., Islam K., Batool F., Tiznobaik M., Hossain F.M.Z., Ahmed K.S., Alam M.S., Ahsan R. (2023). Fiber-reinforced recycled aggregate concrete with crumb rubber: A state-of-the-art review. Constr. Build. Mater..

[29] Malazdrewicz S., Ostrowski K.A., Sadowski Ł. (2023). Self-compacting concrete with recycled coarse aggregates from concrete construction and demolition waste—Current state-of-the art and perspectives. Constr. Build. Mater..

[30] Pepe M., Toledo Filho R.D., Koenders E.A.B., Martinelli E. (2014). Alternative Processing Procedures for Recycled Aggregates in Structural Concrete. Constr. Build. Mater..

[31] Deshpande Y.S., Hiller J.E. (2012). Pore Characterization of Manufactured Aggregates: Recycled Concrete Aggregates and Lightweight Aggregates. Mater Struct.

[32] Fraile-Garcia E., Ferreiro-Cabello J., López-Ochoa L.M., López-González L.M. (2017). Study of the Technical Feasibility of Increasing the Amount of Recycled Concrete Waste Used in Ready-Mix Concrete Production. Materials.

[33] Afroughsabet V., Biolzi L., Ozbakkaloglu T. (2017). Influence of Double Hooked-End Steel Fibers and Slag on Mechanical and Durability Properties of High Performance Recycled Aggregate Concrete. Compos. Struct..

[34] Li L., Xuan D., Sojobi A.O., Liu S., Poon C.S. (2021). Efficiencies of Carbonation and Nano Silica Treatment Methods in Enhancing the Performance of Recycled Aggregate Concrete. Constr. Build. Mater..

[35] Sasanipour H., Aslani F., Taherinezhad J. (2021). Chloride Ion Permeability Improvement of Recycled Aggregate Concrete Using Pretreated Recycled Aggregates by Silica Fume Slurry. Constr. Build. Mater..

[36] Zhang H., Zhao Y. (2016). Performance of Recycled Concrete Beams under Sustained Loads Coupled with Chloride Ion (Cl^−^) Ingress. Constr. Build. Mater..

[37] Zheng Y., Zhuo J., Zhang P. (2021). A Review on Durability of Nano-SiO_2_ and Basalt Fiber Modified Recycled Aggregate Concrete. Constr. Build. Mater..

[38] Al-Bayati H.K.A., Das P.K., Tighe S.L., Baaj H. (2016). Evaluation of Various Treatment Methods for Enhancing the Physical and Morphological Properties of Coarse Recycled Concrete Aggregate. Constr. Build. Mater..

[39] Kong D., Lei T., Zheng J., Ma C., Jiang J., Jiang J. (2010). Effect and Mechanism of Surface-Coating Pozzalanics Materials around Aggregate on Properties and ITZ Microstructure of Recycled Aggregate Concrete. Constr. Build. Mater..

[40] Zhang H., Zhao Y., Meng T., Shah S.P. (2015). The Modification Effects of a Nano-Silica Slurry on Microstructure, Strength, and Strain Development of Recycled Aggregate Concrete Applied in an Enlarged Structural Test. Constr. Build. Mater..

[41] Ho H.-L., Huang R., Lin W.-T., Cheng A. (2018). Pore-Structures and Durability of Concrete Containing Pre-Coated Fine Recycled Mixed Aggregates Using Pozzolan and Polyvinyl Alcohol Materials. Constr. Build. Mater..

[42] Kou S.-C., Poon C.-S. (2010). Properties of Concrete Prepared with PVA-Impregnated Recycled Concrete Aggregates. Cem. Concr. Compos..

[43] Spaeth V., Djerbi Tegguer A. (2013). Improvement of Recycled Concrete Aggregate Properties by Polymer Treatments. Int. J. Sustain. Built Environ..

[44] Tsujino M., Noguchi T., Tamura M., Kanematsu M., Maruyama I. (2007). Application of Conventionally Recycled Coarse Aggregate to Concrete Structure by Surface Modification Treatment. J. Adv. Concr. Technol..

[45] Shaikh F., Chavda V., Minhaj N., Arel H.S. (2018). Effect of Mixing Methods of Nano Silica on Properties of Recycled Aggregate Concrete. Struct. Concr..

[46] Zhang H., Zhao Y., Meng T., Shah S.P. (2016). Surface Treatment on Recycled Coarse Aggregates with Nanomaterials. J. Mater. Civ. Eng..

[47] Zeng W., Zhao Y., Zheng H., Poon C.S. (2020). Improvement in Corrosion Resistance of Recycled Aggregate Concrete by Nano Silica Suspension Modification on Recycled Aggregates. Cem. Concr. Compos..

[48] Poon C.S., Shen P., Jiang Y., Ma Z., Xuan D. (2023). Total Recycling of Concrete Waste Using Accelerated Carbonation: A Review. Cem. Concr. Res..

[49] Wang Y., Liao J., Liu Z. (2023). Service Life Prediction and Environmental Impact Evaluation of Recycled Aggregate Concrete with Supplementary Cementitious Materials. Constr. Build. Mater..

[50] Miller S.A., Srubar W.V., Billington S.L., Lepech M.D. (2015). Integrating Durability-Based Service-Life Predictions with Environmental Impact Assessments of Natural Fiber–Reinforced Composite Materials. Resour. Conserv. Recycl..

[51] Juenger M.C.G., Snellings R., Bernal S.A. (2019). Supplementary Cementitious Materials: New Sources, Characterization, and Performance Insights. Cem. Concr. Res..

[52] Juenger M.C.G., Siddique R. (2015). Recent Advances in Understanding the Role of Supplementary Cementitious Materials in Concrete. Cem. Concr. Res..

[53] Justnes H. How SCMs improve concrete durability—A fundamental view. Proceedings of the Fourth International Conference on Sustainable Construction Materials and Technologies.

[54] Liang C., Cai Z., Wu H., Xiao J., Zhang Y., Ma Z. (2021). Chloride Transport and Induced Steel Corrosion in Recycled Aggregate Concrete: A Review. Constr. Build. Mater..

[55] Ouyang K., Liu J., Liu S., Song B., Guo H., Li G., Shi C. (2023). Influence of Pre-Treatment Methods for Recycled Concrete Aggregate on the Performance of Recycled Concrete: A Review. Resour. Conserv. Recycl..

[56] Shi C., Wu Z., Cao Z., Ling T.C., Zheng J. (2018). Performance of Mortar Prepared with Recycled Concrete Aggregate Enhanced by CO_2_ and Pozzolan Slurry. Cem. Concr. Compos..

[57] Zhang J., Shi C., Li Y., Pan X., Poon C.-S., Xie Z. (2015). Influence of Carbonated Recycled Concrete Aggregate on Properties of Cement Mortar. Constr. Build. Mater..

[58] Zhang H., Zhao Y. (2015). Integrated Interface Parameters of Recycled Aggregate Concrete. Constr. Build. Mater..

[59] Kou S.-C., Poon C.-S. (2013). Long-Term Mechanical and Durability Properties of Recycled Aggregate Concrete Prepared with the Incorporation of Fly Ash. Cem. Concr. Compos..

[60] Wang L., Wang J., Xu Y., Cui L., Qian X., Chen P., Fang Y. (2019). Consolidating Recycled Concrete Aggregates Using Phosphate Solution. Constr. Build. Mater..

[61] Liang C., Pan B., Ma Z., He Z., Duan Z. (2020). Utilization of CO_2_ Curing to Enhance the Properties of Recycled Aggregate and Prepared Concrete: A Review. Cem. Concr. Compos..

[62] Wang L., Wang J., Qian X., Chen P., Xu Y., Guo J. (2017). An Environmentally Friendly Method to Improve the Quality of Recycled Concrete Aggregates. Constr. Build. Mater..

[63] Ahmed W., Lim C.W. (2021). Coupling Effect Assessment of Vacuum Based Pozzolana Slurry Encrusted Recycled Aggregate and Basalt Fiber on Mechanical Performance of Fiber Reinforced Concrete. Constr. Build. Mater..

[64] Ismail S., Ramli M. (2014). Mechanical Strength and Drying Shrinkage Properties of Concrete Containing Treated Coarse Recycled Concrete Aggregates. Constr. Build. Mater..

[65] Santos W.F., Quattrone M., John V.M., Angulo S.C. (2017). Roughness, Wettability and Water Absorption of Water Repellent Treated Recycled Aggregates. Constr. Build. Mater..

[66] Güneyisi E., Gesoğlu M., Algın Z., Yazıcı H. (2014). Effect of Surface Treatment Methods on the Properties of Self-Compacting Concrete with Recycled Aggregates. Constr. Build. Mater..

[67] Shi Z., Lothenbach B., Geiker M.R., Kaufmann J., Leemann A., Ferreiro S., Skibsted J. (2016). Experimental Studies and Thermodynamic Modeling of the Carbonation of Portland Cement, Metakaolin and Limestone Mortars. Cem. Concr. Res..

[68] Zajac M., Skibsted J., Skocek J., Durdzinski P., Bullerjahn F., Ben Haha M. (2020). Phase Assemblage and Microstructure of Cement Paste Subjected to Enforced, Wet Carbonation. Cem. Concr. Res..

[69] Butkute K., Vaitkevicius V., Sinka M., Augonis A., Korjakins A. (2023). Influence of Carbonated Bottom Slag Granules in 3D Concrete Printing. Materials.

[70] Zhan B.J., Xuan D.X., Poon C.S. (2018). Enhancement of Recycled Aggregate Properties by Accelerated CO_2_ Curing Coupled with Limewater Soaking Process. Cem. Concr. Compos..

[71] Gholizadeh-Vayghan A., Bellinkx A., Snellings R., Vandoren B., Quaghebeur M. (2020). The Effects of Carbonation Conditions on the Physical and Microstructural Properties of Recycled Concrete Coarse Aggregates. Constr. Build. Mater..

[72] Zhan B.J., Xuan D.X., Zeng W., Poon C.S. (2019). Carbonation Treatment of Recycled Concrete Aggregate: Effect on Transport Properties and Steel Corrosion of Recycled Aggregate Concrete. Cem. Concr. Compos..

[73] Angst U.M. (2023). Steel Corrosion in Concrete—Achilles’ Heel for Sustainable Concrete?. Cem. Concr. Res..

[74] Kou S.-C., Zhan B., Poon C.-S. (2014). Use of a CO_2_ Curing Step to Improve the Properties of Concrete Prepared with Recycled Aggregates. Cem. Concr. Compos..

[75] Monkman S., Shao Y. (2010). Carbonation Curing of Slag-Cement Concrete for Binding CO_2_ and Improving Performance. J. Mater. Civ. Eng..

[76] Kashef-Haghighi S., Ghoshal S. (2010). CO_2_ Sequestration in Concrete through Accelerated Carbonation Curing in a Flow-through Reactor. Ind. Eng. Chem. Res..

[77] Fang X., Xuan D., Poon C.S. (2017). Empirical Modelling of CO_2_ Uptake by Recycled Concrete Aggregates under Accelerated Carbonation Conditions. Mater Struct.

[78] Zhan B.J., Poon C.S., Shi C.J. (2016). Materials Characteristics Affecting CO_2_ Curing of Concrete Blocks Containing Recycled Aggregates. Cem. Concr. Compos..

[79] Lim M., Han G.-C., Ahn J.-W., You K.-S. (2010). Environmental Remediation and Conversion of Carbon Dioxide (CO_2_) into Useful Green Products by Accelerated Carbonation Technology. Int. J. Environ. Res. Public Health.

[80] Zajac M., Skibsted J., Bullerjahn F., Skocek J. (2022). Semi-Dry Carbonation of Recycled Concrete Paste. J. CO2 Util..

[81] Wu Y., Mehdizadeh H., Mo K.H., Ling T.-C. (2022). High-Temperature CO_2_ for Accelerating the Carbonation of Recycled Concrete Fines. J. Build. Eng..

[82] De Juan M.S., Gutiérrez P.A. (2009). Study on the Influence of Attached Mortar Content on the Properties of Recycled Concrete Aggregate. Constr. Build. Mater..

[83] Akbarnezhad A., Ong K.C.G., Zhang M.H., Tam C.T., Foo T.W.J. (2011). Microwave-Assisted Beneficiation of Recycled Concrete Aggregates. Constr. Build. Mater..

[84] Toshio Y., Yukio K., Kunio Y., Masaro K., Kazuaki A., Masaru Y. (2001). A study on a technology for producing high quality recycled coarse aggregate. Materials.

[85] Yoda K., Harada M., Sakuramoto F. (2015). Field application and advantage of concrete recycled in-situ recycling systems. Recycling and Reuse of Waste Materials.

[86] Paul S.C. (2017). Data on Optimum Recycle Aggregate Content in Production of New Structural Concrete. Data Brief.

[87] Shima H., Tateyashiki H., Matsuhashi R., Yoshida Y. (2005). An Advanced Concrete Recycling Technology and Its Applicability Assessment through Input-Output Analysis. J. Adv. Concr. Technol..

[88] Lippiatt N., Bourgeois F. (2012). Investigation of Microwave-Assisted Concrete Recycling Using Single-Particle Testing. Miner. Eng..

[89] Bru K., Touzé S., Bourgeois F., Lippiatt N., Ménard Y. (2014). Assessment of a Microwave-Assisted Recycling Process for the Recovery of High-Quality Aggregates from Concrete Waste. Int. J. Miner. Process..

[90] Tam V.W.Y., Tam C.M., Le K.N. (2007). Removal of Cement Mortar Remains from Recycled Aggregate Using Pre-Soaking Approaches. Resour. Conserv. Recycl..

[91] Katkhuda H., Shatarat N. (2017). Improving the Mechanical Properties of Recycled Concrete Aggregate Using Chopped Basalt Fibers and Acid Treatment. Constr. Build. Mater..

[92] Alvi I.H., Li Q., Hou Y., Onyekwena C.C., Zhang M., Ghaffar A. (2023). A Critical Review of Cement Composites Containing Recycled Aggregates with Graphene Oxide Nanomaterials. J. Build. Eng..

[93] Kong D., Huang S., Corr D., Yang Y., Shah S.P. (2018). Whether Do Nano-Particles Act as Nucleation Sites for C-S-H Gel Growth during Cement Hydration?. Cem. Concr. Compos..

[94] Ababneh A., Benboudjema F., Xi Y. (2003). Chloride Penetration in Nonsaturated Concrete. J. Mater. Civ. Eng..

[95] Oh B.H., Jang S.Y. (2007). Effects of Material and Environmental Parameters on Chloride Penetration Profiles in Concrete Structures. Cem. Concr. Res..

[96] Alessandro Pansera W., Alessi Reichert T., Savaris G., Eduardo Tino Balestra C. (2022). Nonlinear Regression Using Gaussian-Lorentzian Functions to Empirical Modeling of Convective-Diffusive Chloride Transport in Concrete. Constr. Build. Mater..

[97] Jensen O.M., Coats A.M., Glasser F.P. (1996). Chloride Ingress Profiles Measured by Electron Probe Micro Analysis. Cem. Concr. Res..

[98] Chatterji S. (1995). On the Applicability of Fick’s Second Law to Chloride Ion Migration through Portland Cement Concrete. Cem. Concr. Res..

[99] Goldman A., Bentur A. (1993). The Influence of Microfillers on Enhancement of Concrete Strength. Cem. Concr. Res..

[100] Wang J., Zhang J., Cao D., Dang H., Ding B. (2020). Comparison of Recycled Aggregate Treatment Methods on the Performance for Recycled Concrete. Constr. Build. Mater..

[101] Zhang J., Wang J., Li X., Zhou T., Guo Y. (2018). Rapid-Hardening Controlled Low Strength Materials Made of Recycled Fine Aggregate from Construction and Demolition Waste. Constr. Build. Mater..

[102] Zhang H., Ji T., Liu H., Su S. (2018). Modifying Recycled Aggregate Concrete by Aggregate Surface Treatment Using Sulphoaluminate Cement and Basalt Powder. Constr. Build. Mater..

[103] Zhang H., Liu W., Lin X., Su S., Zhao B. (2021). To Ameliorate the Performance of Recycled Aggregate Concrete (RAC) by Pre-Treating Aggregate in Sulfoaluminate Cement Slurry and Water Glass Solution. J. Build. Eng..

[104] Li X., Li R., Liu X. (2011). The influence of sodium silicate on the impermeability of cement. J. Qiqihar Univ..

[105] Shi C., Li Y., Zhang J., Li W., Chong L., Xie Z. (2016). Performance Enhancement of Recycled Concrete Aggregate—A Review. J. Clean. Prod..

[106] Sari M., Prat E., Labastire J.-F. (1999). High Strength Self-Compacting Concrete Original Solutions Associating Organic and Inorganic Admixtures. Cem. Concr. Res..

[107] Senff L., Labrincha J.A., Ferreira V.M., Hotza D., Repette W.L. (2009). Effect of Nano-Silica on Rheology and Fresh Properties of Cement Pastes and Mortars. Constr. Build. Mater..

[108] Zhu Y.-G., Kou S.-C., Poon C.-S., Dai J.-G., Li Q.-Y. (2013). Influence of Silane-Based Water Repellent on the Durability Properties of Recycled Aggregate Concrete. Cem. Concr. Compos..

[109] Xuan D., Zhan B., Poon C.S. (2017). Durability of Recycled Aggregate Concrete Prepared with Carbonated Recycled Concrete Aggregates. Cem. Concr. Compos..

[110] Sasanipour H., Aslani F., Taherinezhad J. (2019). Effect of Silica Fume on Durability of Self-Compacting Concrete Made with Waste Recycled Concrete Aggregates. Constr. Build. Mater..

[111] Kim Y., Hanif A., Kazmi S.M.S., Munir M.J., Park C. (2018). Properties Enhancement of Recycled Aggregate Concrete through Pretreatment of Coarse Aggregates—Comparative Assessment of Assorted Techniques. J. Clean. Prod..

[112] Kazmi S.M.S., Munir M.J., Wu Y.-F., Patnaikuni I., Zhou Y., Xing F. (2020). Effect of Recycled Aggregate Treatment Techniques on the Durability of Concrete: A Comparative Evaluation. Constr. Build. Mater..

[113] Ismail S., Ramli M. (2013). Engineering Properties of Treated Recycled Concrete Aggregate (RCA) for Structural Applications. Constr. Build. Mater..

[114] Sim J., Park C. (2011). Compressive Strength and Resistance to Chloride Ion Penetration and Carbonation of Recycled Aggregate Concrete with Varying Amount of Fly Ash and Fine Recycled Aggregate. Waste Manag..

[115] Kim K., Shin M., Cha S. (2013). Combined Effects of Recycled Aggregate and Fly Ash towards Concrete Sustainability. Constr. Build. Mater..

[116] Kou S., Poon C., Agrela F. (2011). Comparisons of Natural and Recycled Aggregate Concretes Prepared with the Addition of Different Mineral Admixtures. Cem. Concr. Compos..

[117] Ann K.Y., Moon H.Y., Kim Y.B., Ryou J. (2008). Durability of Recycled Aggregate Concrete Using Pozzolanic Materials. Waste Manag..

[118] Qi B., Gao J., Chen F., Shen D. (2018). Chloride Penetration into Recycled Aggregate Concrete Subjected to Wetting–Drying Cycles and Flexural Loading. Constr. Build. Mater..

[119] Kurda R., Silvestre J.D., De Brito J., Ahmed H. (2018). Optimizing Recycled Concrete Containing High Volume of Fly Ash in Terms of the Embodied Energy and Chloride Ion Resistance. J. Clean. Prod..

[120] Chen A.J., Wang J., Ge Z.F., Wu M. (2011). Permeability of Anti-Chloride Ion of Fly Ash Recycled Concrete. Adv. Mater. Res..

[121] Qin H.Y., Zhao Y.L., Luo B.G., Chen Y.H. (2010). Experimental Study on the Chloride Diffusivity of Recycled Aggregate Concrete. Adv. Mater. Res..

[122] Kapoor K., Singh S.P., Singh B. (2020). Evaluating the Durability Properties of Self Compacting Concrete Made with Coarse and Fine Recycled Concrete Aggregates. Eur. J. Environ. Civ. Eng..

[123] Chindaprasirt P., Rukzon S., Sirivivatnanon V. (2008). Effect of Carbon Dioxide on Chloride Penetration and Chloride Ion Diffusion Coefficient of Blended Portland Cement Mortar. Constr. Build. Mater..

[124] Berndt M.L. (2009). Properties of Sustainable Concrete Containing Fly Ash, Slag and Recycled Concrete Aggregate. Constr. Build. Mater..

[125] Kapoor K., Singh S.P., Singh B. (2016). Durability of Self-Compacting Concrete Made with Recycled Concrete Aggregates and Mineral Admixtures. Constr. Build. Mater..

[126] Koushkbaghi M., Kazemi M.J., Mosavi H., Mohseni E. (2019). Acid Resistance and Durability Properties of Steel Fiber-Reinforced Concrete Incorporating Rice Husk Ash and Recycled Aggregate. Constr. Build. Mater..

[127] Alnahhal M.F., Alengaram U.J., Jumaat M.Z., Abutaha F., Alqedra M.A., Nayaka R.R. (2018). Assessment on Engineering Properties and CO_2_ Emissions of Recycled Aggregate Concrete Incorporating Waste Products as Supplements to Portland Cement. J. Clean. Prod..

[128] Rattanachu P., Toolkasikorn P., Tangchirapat W., Chindaprasirt P., Jaturapitakkul C. (2020). Performance of Recycled Aggregate Concrete with Rice Husk Ash as Cement Binder. Cem. Concr. Compos..

[129] Rattanachu P., Karntong I., Tangchirapat W., Jaturapitakkul C., Chindaprasirt P. (2018). Influence of Bagasse Ash and Recycled Concrete Aggregate on Hardened Properties of High-Strength Concrete. Mater. Construcc..

[130] Somna R., Jaturapitakkul C., Amde A.M. (2012). Effect of Ground Fly Ash and Ground Bagasse Ash on the Durability of Recycled Aggregate Concrete. Cem. Concr. Compos..

[131] Amiri M., Hatami F., Golafshani E.M. (2021). Evaluating the Synergic Effect of Waste Rubber Powder and Recycled Concrete Aggregate on Mechanical Properties and Durability of Concrete. Case Stud. Constr. Mater..

[132] Guo M., Gong G., Yue Y., Xing F., Zhou Y., Hu B. (2022). Performance Evaluation of Recycled Aggregate Concrete Incorporating Limestone Calcined Clay Cement (LC3). J. Clean. Prod..

[133] Ying J., Zhou B., Xiao J. (2017). Pore Structure and Chloride Diffusivity of Recycled Aggregate Concrete with Nano-SiO_2_ and Nano-TiO_2_. Constr. Build. Mater..

[134] Ali B., Qureshi L.A. (2019). Influence of Glass Fibers on Mechanical and Durability Performance of Concrete with Recycled Aggregates. Constr. Build. Mater..

[135] Dash B., Prakash Giri J., Markandeya Raju P., Dora D.T.K. (2023). Simultaneous Influence of Processed Cellulose Acetate Fiber Reinforcement and Recycled Aggregate Replacement on Mechanical and Durability Performances of Concrete. Constr. Build. Mater..

[136] Ma Z., Liu M., Tang Q., Liang C., Duan Z. (2020). Chloride Permeability of Recycled Aggregate Concrete under the Coupling Effect of Freezing-Thawing, Elevated Temperature or Mechanical Damage. Constr. Build. Mater..

[137] (2009). Standard for Test Method of Long-Term Performance and Durability of Ordinary Concrete.

[138] (1999). Concrete, Mortar and Cement-Based Repair Materials: Chloride Migration Coefficient form Non-Steady-State Migration Experiments.

[139] Bao J., Li S., Zhang P., Ding X., Xue S., Cui Y., Zhao T. (2020). Influence of the Incorporation of Recycled Coarse Aggregate on Water Absorption and Chloride Penetration into Concrete. Constr. Build. Mater..

[140] Tuyan M., Mardani-Aghabaglou A., Ramyar K. (2014). Freeze–Thaw Resistance, Mechanical and Transport Properties of Self-Consolidating Concrete Incorporating Coarse Recycled Concrete Aggregate. Mater. Des..

[141] (1997). Standard Test Method for Electrical Indication of Concrete’s Ability to Resist Chloride Ion Penetration.

[142] Thomas M.D.A., Jones M.R. (1996). A Critical Review of Service Life Modelling of Concretes Exposed to Chlorides. Concrete in the Service of Mankind.

[143] Silva S., Evangelista L., De Brito J. (2021). Durability and Shrinkage Performance of Concrete Made with Coarse Multi-Recycled Concrete Aggregates. Constr. Build. Mater..

[144] Amorim Júnior N.S., Silva G.A.O., Dias C.M.R., Ribeiro D.V. (2019). Concrete Containing Recycled Aggregates: Estimated Lifetime Using Chloride Migration Test. Constr. Build. Mater..

[145] Pedro D., De Brito J., Evangelista L. (2014). Influence of the Use of Recycled Concrete Aggregates from Different Sources on Structural Concrete. Constr. Build. Mater..

[146] Chakradhara Rao M., Bhattacharyya S.K., Barai S.V. (2011). Influence of Field Recycled Coarse Aggregate on Properties of Concrete. Mater. Struct..

[147] Kou S., Poon C. (2015). Effect of the Quality of Parent Concrete on the Properties of High Performance Recycled Aggregate Concrete. Constr. Build. Mater..

[148] Andreu G., Miren E. (2014). Experimental Analysis of Properties of High Performance Recycled Aggregate Concrete. Constr. Build. Mater..

[149] Gebremariam H.G., Taye S., Tarekegn A.G. (2023). Disparity in Research Findings on Parent Concrete Strength Effects on Recycled Aggregate Quality as a Challenge in Aggregate Recycling. Case Stud. Constr. Mater..

[150] Gholampour A., Ozbakkaloglu T. (2018). Time-Dependent and Long-Term Mechanical Properties of Concretes Incorporating Different Grades of Coarse Recycled Concrete Aggregates. Eng. Struct..

[151] Duan Z., Han N., Singh A., Xiao J. (2020). Multi-Scale Investigation on Concrete Prepared with Recycled Aggregates from Different Parent Concrete. J. Renew. Mater..

[152] Chakradhara Rao M. (2018). Properties of Recycled Aggregate and Recycled Aggregate Concrete: Effect of Parent Concrete. Asian J. Civ. Eng..

[153] Zhu P., Hao Y., Liu H., Wei D., Liu S., Gu L. (2019). Durability Evaluation of Three Generations of 100% Repeatedly Recycled Coarse Aggregate Concrete. Constr. Build. Mater..

[154] Zhu P., Liu W., Niu Z., Wei D., Hu K. (2018). Strength and Chloride Diffusion Behaviour of Three Generations of Repeated Recycled Fine Aggregate Concrete. J. Wuhan Univ. Technol.-Mater. Sci. Ed..

[155] Amorim Júnior N.S., Silva G.A.O., Ribeiro D.V. (2018). Effects of the Incorporation of Recycled Aggregate in the Durability of the Concrete Submitted to Freeze-Thaw Cycles. Constr. Build. Mater..

[156] Butler L., West J.S., Tighe S.L. (2011). The Effect of Recycled Concrete Aggregate Properties on the Bond Strength between RCA Concrete and Steel Reinforcement. Cem. Concr. Res..

[157] Hao L., Liu Y., Wang W., Zhang J., Zhang Y. (2018). Effect of Salty Freeze-Thaw Cycles on Durability of Thermal Insulation Concrete with Recycled Aggregates. Constr. Build. Mater..

[158] Peng G.F., Shang Y.J., Wang Y.J. (2014). Effect of Loading on Permeability of Recycled Aggregate Concrete. Key Eng. Mater..

[159] Wenjian W., Jin W., Zhe W., Guanzheng W., Anyi Y. (2016). Chloride Diffusion Coefficient of Recycled Aggregate Concrete under Compressive Loading. Mater. Struct..

[160] Wang H., Sun X., Wang J., Monteiro P. (2016). Permeability of Concrete with Recycled Concrete Aggregate and Pozzolanic Materials under Stress. Materials.

[161] Luping T., Nilsson L.-O. (1993). Chloride Binding Capacity and Binding Isotherms of OPC Pastes and Mortars. Cem. Concr. Res..

[162] Delagrave A., Marchand J., Ollivier J.-P., Hazrati K. (1997). Chloride Binding Capacity of Various Hydrated Cement Paste Systems. Adv. Cem. Based Mater..

[163] Xiao J., Ying J., Tam V.W.Y., Gilbert I.R. (2014). Test and Prediction of Chloride Diffusion in Recycled Aggregate Concrete. Sci. China Technol. Sci..

[164] Bai G., Zhu C., Liu C., Liu H. (2019). Chloride Ion Invasive Behavior of Recycled Aggregate Concrete under Coupling Flexural Loading and Wetting-Drying Cycles. KSCE J. Civ. Eng..

[165] Alexander M., Beushausen H. (2019). Durability, Service Life Prediction, and Modelling for Reinforced Concrete Structures—Review and Critique. Cem. Concr. Res..

[166] Chen A.J., Wang J., Ge Z.F., Wu M. (2011). The Durability Life Prediction of Recycled Concrete under Chlorate Environment. Adv. Mater. Res..

[167] Jin L., Dong T., Fan T., Duan J., Yu H., Jiao P., Zhang W. (2022). Prediction of the Chloride Diffusivity of Recycled Aggregate Concrete Using Artificial Neural Network. Mater. Today Commun..

[168] (2004). Guide to Durability Design and Construction of Concrete Structures.

[169] Castellote M., Andrade C., Alonso C. (2001). Measurement of the Steady and Non-Steady-State Chloride Diffusion Coefficients in a Migration Test by Means of Monitoring the Conductivity in the Anolyte Chamber. Comp. Nat. Diffus. Tests. Cem. Concr. Res..

[170] Albuquerque A., Pacheco J., Brito J. (2021). Eurocode Design of Recycled Aggregate Concrete for Chloride Environments: Stochastic Modeling of Chloride Migration and Reliability-Based Calibration of Cover. Crystals.

[171] Ying J., Xiao J., Meng Q. (2016). On Probability Distribution of Chloride Diffusion Coefficient for Recycled Aggregate Concrete. Int. J. Concr. Struct. Mater..

[172] FIB (2006). Model Code for Service Life Design.

[173] Stambaugh N.D., Bergman T.L., Srubar W.V. (2018). Numerical Service-Life Modeling of Chloride-Induced Corrosion in Recycled-Aggregate Concrete. Constr. Build. Mater..

